# Aza-proline effectively mimics l-proline stereochemistry in triple helical collagen†
†Electronic supplementary information (ESI) available: A detailed explanation of all procedures for synthesis, purification, characterization, crystallography, and computational analysis. See DOI: 10.1039/c9sc02211b


**DOI:** 10.1039/c9sc02211b

**Published:** 2019-06-21

**Authors:** Alexander J. Kasznel, Trevor Harris, Nicholas J. Porter, Yitao Zhang, David M. Chenoweth

**Affiliations:** a Department of Chemistry , University of Pennsylvania , 231 S. 34th St. , Philadelphia , PA 19104-6323 , USA . Email: dcheno@sas.upenn.edu; b Department of Bioengineering , University of Pennsylvania , 210 S. 33rd St. , Philadelphia , PA 19104-6323 , USA

## Abstract

The prevalence of l-amino acids in biomolecules has been shown to have teleological importance in biomolecular structure and self-assembly. Recently, biophysical studies have demonstrated that natural l-amino acids can be replaced with non-natural achiral aza-amino acids in folded protein structures such as triple helical collagen. However, the structural consequences of achiral aza-amino acid incorporation has not been elucidated in the context of any relevant folded biomolecule. Herein, we use X-ray crystallography to provide the first atomic resolution crystal structure of an achiral aza-amino acid residue embedded within a folded protein structure, definitively illustrating that achiral aza-proline has the capacity to effectively mimic the stereochemistry of natural amino acids within the context of triple helical collagen. We further corroborate this finding with density functional theory computational analysis showing that the natural l-amino acid stereochemistry for aza-proline is energetically favored when arranged in the aza-proline-hydroxyproline-glycine motif. In addition to providing fundamental insight into peptide and protein structure, the incorporation of achiral stereochemical mimics such as aza-amino acids could have far reaching impacts in areas ranging from synthetic materials to drug design.

## Abstract 2

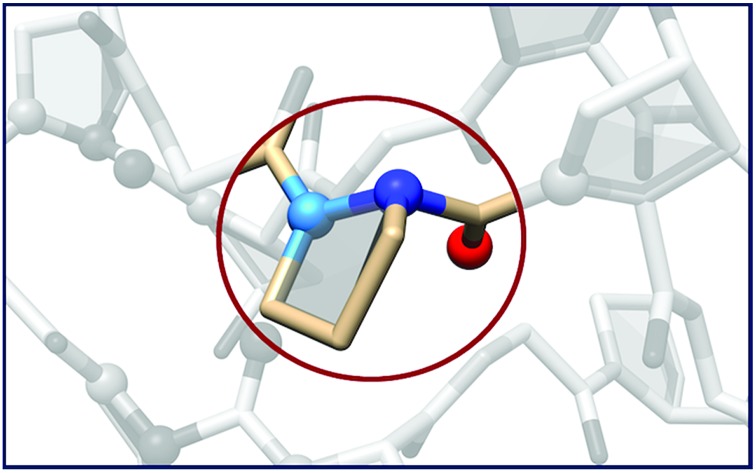
Chenoweth and co-workers provide an atomic resolution crystal structure and computational analysis illustrating that aza-proline mimics l-proline stereochemistry in collagen.

## Introduction

A prevalent feature of structural biology is the “monochirality of life,” best exemplified by the ubiquity of isotactic polymers in biological systems.^1^ In nature, both proteins and nucleic acids are predominately homochiral. This property has been shown to facilitate biomolecular interaction and also enable expedient protein folding by lowering the entropy of biomolecular assembly.1b,^2^ The chirality of life's amino acid building blocks is thus a crucial factor in the synthesis and stability of essential biomolecules.

Collagen is a critical structural protein in mammals. The principal characteristics of collagen are its triple helical structure and triplet repeat amino acid sequence (XaaYaaGly)_*n*_; the Xaa and Yaa positions are typically filled by proline (Pro, P) and the post-translationally modified amino acid hydroxyproline (Hyp, O), respectively.^3^ Our lab has investigated the structure and function of collagen by synthesizing an array of collagen model peptides with novel biophysical properties, most notably by integrating the non-natural amino acids aza-glycine (azGly, azG)^4^ and aza-proline (azPro, azP).^5^ These aza-peptides have the α-CH group from one or more amino acid residues in their peptide chain replaced with a N atom.^6^ This seemingly small structural change can have substantial effects on peptide structure and electronics such as increasing intrinsic chemical stability and resistance towards biodegradation^7^ and improving collagen stability and self-assembly.4b,c These properties have led to the pursuit of aza-peptides as promising candidates for therapeutic applications,^8^ which generated a wave of new solid-phase synthetic methods^9^ and fundamental studies.^10^ Collagen peptides containing non-natural amino acid substitutions that do not disturb the overall collagen topology would be useful biological tools to probe the collagen interactome.3*b*

**Fig. 1:**
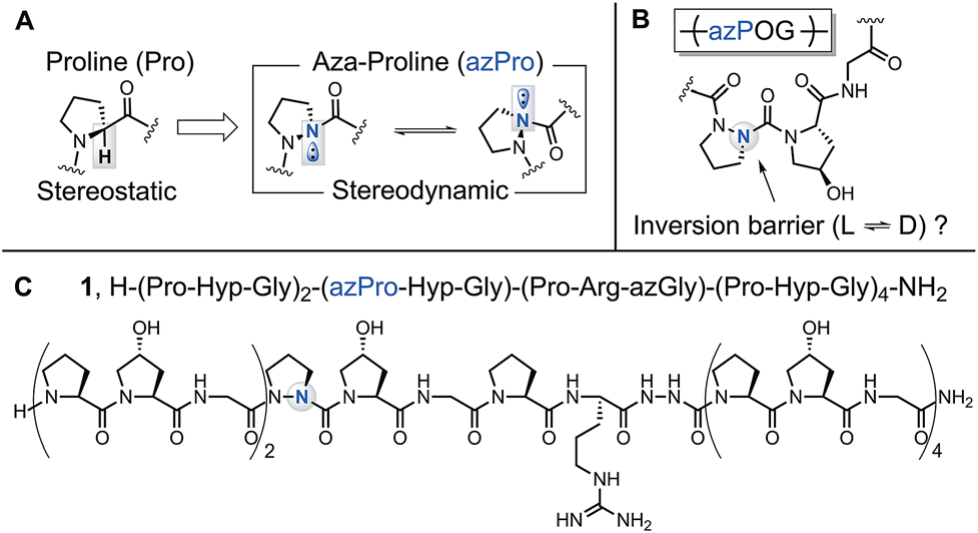
Synthetic modification to collagen peptide backbone using azPro. (A) Backbone structures of Pro and azPro illustrating stereodynamic N atom in azPro. (B) Inversion of azPro in the context of an azPOG triplet in collagen. (C) Backbone structure of CMP **1** showing site of azPro substitution.

The added nitrogen atom in azPro is notable for its sp^3^-like pyramidalized conformation (Fig. 1A).10a,^11^ Although the alteration of the α-CH stereocenter in Pro to N results in a net isoelectronic change, this modification has a large effect on the conformational properties of the α-stereocenter and gives the azPro ring the ability to mimic either d- or l-amino acid stereochemistry. Unlike electronically neutral carbon, nitrogen has a lone pair that can rehybridize during pyramidal inversion, turning this inversion into a dynamic process.^12^

In a previous study of a collagen model peptide (CMP), the added stereodynamic center in azPro slowed the rate of triple helical self-assembly.^5^ However, because azPro is typically studied in the context of short β-turn mimics,10a,f,^11^,^13^ many details of azPro's structural behavior in collagen remain uncharacterized: (1) Does the added N atom in azPro pyramidalize in collagen? If so, does the triple helical structure force azPro's pyramidal conformation, or do the underpinning steric and conformational constraints of the azPro backbone play a greater role in determining this? (2) Does azPro mimic the conformation of the canonical Pro residue in collagen? (3) Finally, what is the barrier to azPro's pyramidal inversion in the context of a collagen amino acid sequence (Fig. 1B)?

To answer these questions, we synthesized azPro-containing CMP **1**, H–(POG)_2_(azPOG)(PRazG)(POG)_4_–NH_2_ (Fig. 1C), and determined its structure using X-ray crystallography. We also performed density functional theory (DFT) calculations to elucidate azPro's stereodynamic nature in a collagen peptide. To date, there have been few published examples of azPro in the crystal state.11a,^14^ Notably, none of these have shown azPro in the context of any biologically relevant macromolecule or higher-order molecular assembly. In this publication, we present the first atomic resolution crystal structure of collagen containing azPro, and our findings indicate that azPro is a structural mimic of its natural amino acid counterpart in collagen.

## Results and discussion

### Crystallography

**Fig. 2:**
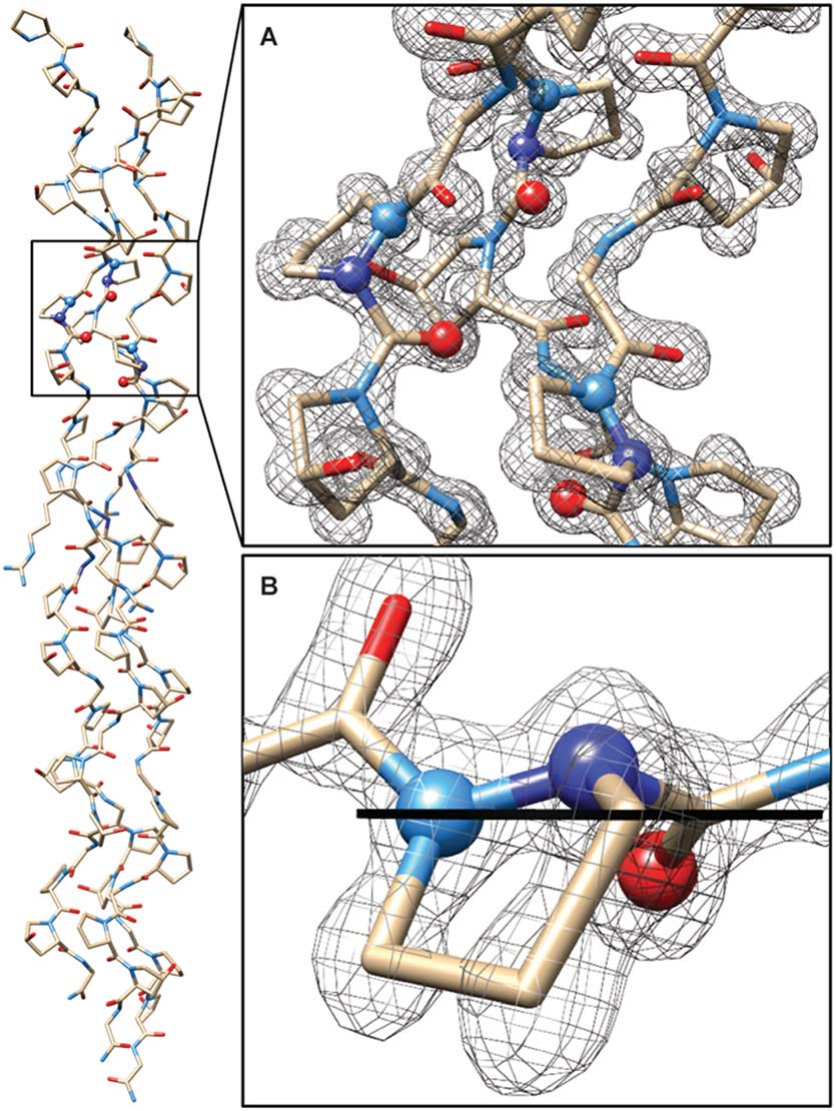
Crystal structure of CMP **1** solved to 1.10 Å resolution. (Left) Structure confirming formation of triple helix (*R*_work_ = 11.7%, *R*_free_ = 14.4%, *P*2_1_). (A) Magnified view of triple helix with electron density at site of azPro integration (2mFo-DFc map contoured at 1.8*σ*). (B) Close-up of single azPro residue with plane bisecting atoms connected to pyramidalized nitrogen. Light blue = nitrogens already present in collagen, dark blue = added nitrogen in azPro.

In CMP **1**, one Pro residue from each strand was replaced with its aza-derivative azPro (Fig. 1C). This amino acid sequence was engineered to produce a homotrimeric peptide containing a total of 3 azPro residues when fully assembled. CMP **1** was synthesized on solid phase and crystallized by sitting-drop vapor diffusion (detailed procedures in ESI†). The crystal structure of **1** (PDB ; 6M80) reveals that this CMP still forms a triple helix despite the introduction of azPro to its backbone and that azPro does adopt a pyramidal configuration within this helix (Fig. 2). This structural evidence illustrates that azPro is not held in a quasiplanar state and that the superseding energetics of the collagen assembly could compensate for this destabilized azPro configuration.

Since the dominating structural framework and energetics of the triple helix can theoretically hold azPro in either a quasiplanar or pyramidal configuration, we investigated whether the overall triple helical structure or azPro's fundamental backbone architecture were responsible for azPro's pyramidal configuration. To determine this, we examined 26 unique structures from the Cambridge Structural Database (CSD) containing *N*-amidourea moieties of the general form RN_*x*_(O)C–N_*y*_R–N_*z*_R–C(O)R (where N_*y*_ is the pyramidal atom in azPro) in their respective backbones (see Fig. S1–S3 and Table S1†). We observed an increase in pyramidalization in the N_*y*_ atom position as the degree of substitution increased at the N_*x*_, N_*y*_, and N_*z*_ atoms. Pyramidalization becomes more pronounced at the N_*y*_ atom when it is incorporated into a cycle as it is in azPro. Integrating N_*y*_ into a cycle creates steric and conformational constraints on the conjugated amide framework by restricting the main-chain phi dihedral (C–N_*y*_–N_*z*_–C). This leads the system to balance several structural and electronic factors such as amide resonance, ring strain, and sterics. As a result, amide resonance is weakened in azPro, the effects of which can be seen in its non-planar structure and long amide N–CO bonds. The pyramidal N sites of the three azPro rings in the triple helix of CMP **1** have N–CO bond distances of 1.42, 1.44, and 1.48 Å, longer than a typical tertiary amide bond length of ∼1.34 Å.^15^ This indicates that azPro's pyramidalization is connected to the underlying functionality in its backbone rather than the long-range forces of the triple helix.

The combined structural distortions observed in the azPro residues also resemble the features of classical twisted amides (Table S2†).^16^ However, because of delocalization throughout the conjugated amide framework, azPro residues do not exhibit the same high reactivity as classical twisted amides. The extended conjugated backbone provides azPro with increased resonance stabilization although, due to the aforementioned structural distortions (pyramidalization and twisting), resonance is slightly weakened.

**Fig. 3:**
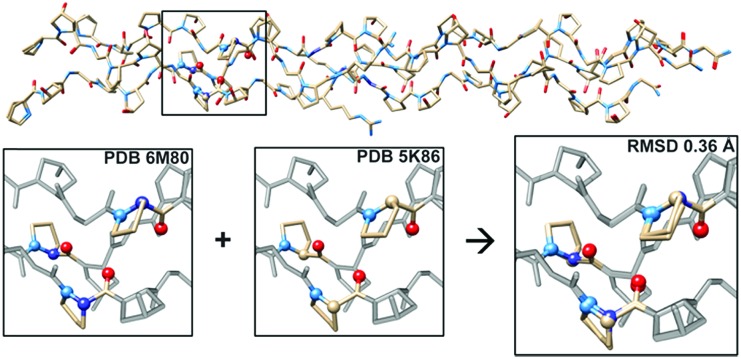
Aza-proline mimics proline in backbone of CMP. (Top) Superposition of crystal structures of CMPs **1** and **2** showing close matching of backbone and side chain conformations throughout; RMSD = 0.36 Å. (Bottom) Close-up of boxed region in **1** (PDB ; 6M80), **2** (PDB ; 5K86), and overlay of the two structures, illustrating correspondence between conformation of azPro7 in **1** and Pro7 in **2**.

To further investigate the structural implications of azPro substitution in collagen, we compared the crystal structure of CMP **1** to that of previously published triple-helical CMP **2**, H–(POG)_3_(PRazG)(POG)_4_–NH_2_, which contains Pro instead of azPro.4*a* This comparison indicates an overall RMSD of 0.36 Å for all atoms (468 atom pairs; H atoms excluded), and more specifically reveals that the azPro residues in **1** adopt the same conformation as the corresponding Pro residues in **2** (Fig. 3).

The angles around the pyramidal N sites of the azPro residues in CMP **1** are slightly shallower than the angles around the tetrahedral C sites of the corresponding canonical Pro residues in CMP **2** (Tables S3 and S4†). Taken together, these data indicate that CMPs containing azPro can still self-assemble into the biologically essential collagen triple helix and that this modification does not disturb the conformations of the peptide backbone or amino acid side chains.

### Computational analysis

**Fig. 4:**
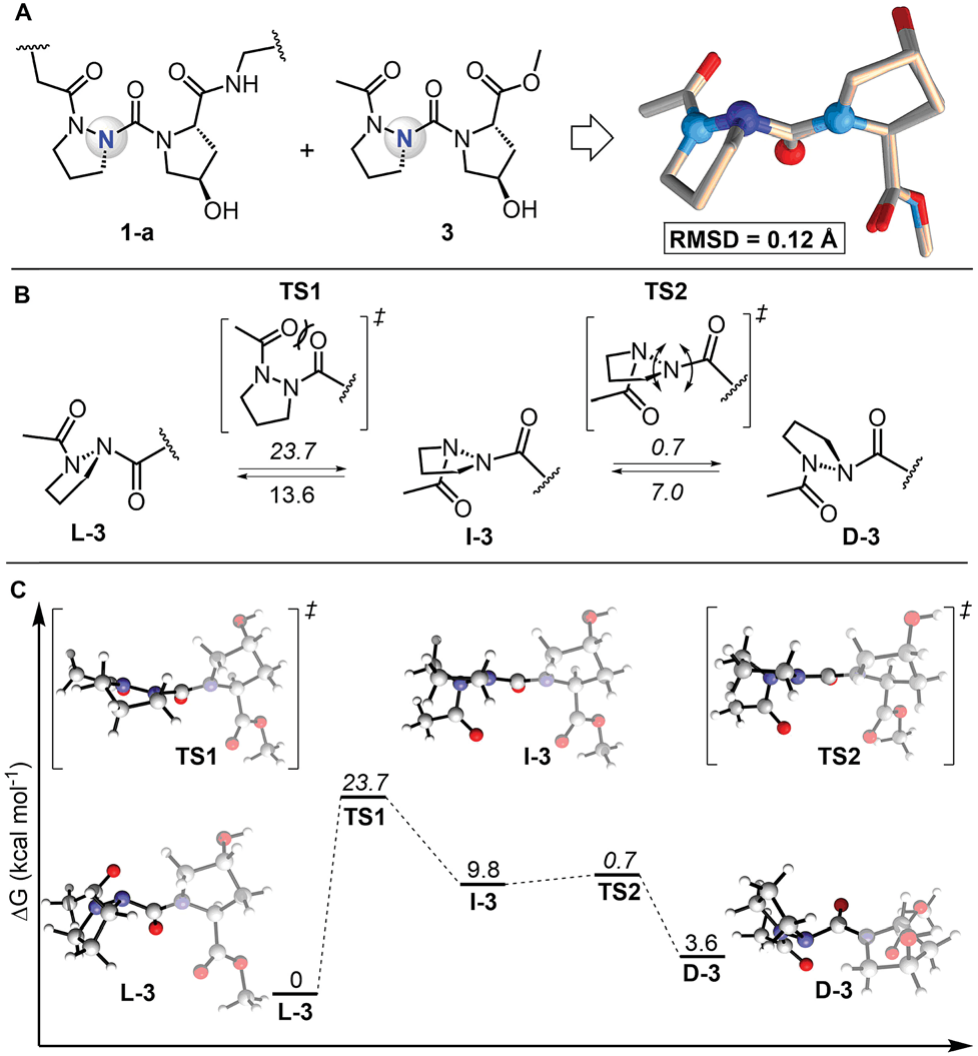
Comparison of calculated model system with experimental collagen fragment and free energy profile for interconversion of backbone N atom in azPro. (A) Overlay of azPro-Hyp-Gly crystal structure fragment (PDB 6M80) and model dipeptide system Ac-azPro-Hyp-OMe (**3**). **3** was calculated using M06-2X/6-31+G(d,p)/SMD = H_2_O. 20 atom pairs were used for RMSD; H atoms were excluded. (B) Pyramidal inversion of azPro in context of **3**. (C) Free energy profile for inversion of azPro. Energies are relative to **L-3** except for barriers (italicized), which are relative to preceding minima.

To study the self-assembly of this azPro-containing CMP, we characterized the stereodynamic nature of azPro using DFT. We first compared the crystal structure fragment azPro-Hyp-Gly (**1-a**) and the low-energy conformer of the dipeptide model system Ac-azPro-Hyp-OMe (**3**).^5^ The overlay of these two structures reveals that their conformations match closely, with an overall RMSD of 0.12 Å (Fig. 4A). This statistical analysis leads us to conclude that azPro readily adopts the conformation necessary for collagen self-assembly rather than being forced into a pyramidal conformation by the overall triple helical structure of the peptide strands. Since the calculated collagen model system mimics the azPro-Hyp-Gly fragment from the collagen triple helix accurately, we used it to determine the energetic cost for pyramidal inversion of azPro. Pyramidal inversion of amines typically proceeds through one planar transition state in which nitrogen's bonding orbitals are ∼sp^2^ hybridized and its lone pair occupies a p orbital.^17^ Conversely, we found that the overall inversion of azPro (**L-3** to **D-3**, where the prefixes indicate the stereochemical notation for azPro) is composed of two transition states connected by intermediate **I-3** (Fig. 4B). The first transition state **TS1** involves steric repulsion between the central carbonyl group, which connects the Hyp ring to the azPro ring, and the acyl carbonyl group as the two cross paths. The distance between the two oxygen atoms of their respective carbonyls in the transition state (2.7 Å) is smaller than the sum of their van der Waals radii (The van der Waals radius of oxygen is 1.52 Å). This barrier, >20 kcal mol^–1^, also reflects the endergonic nature of **I-3**, which is almost 10 kcal mol^–1^ higher than **L-3**.

The second transition state **TS2**, with an activation barrier of only ∼1 kcal mol^–1^, returns the quasi-planar azPro to a completely pyramidal geometry in **D-3**. The free energy profile for the inversion pathway reveals a conformational bias as **D-3** is ∼4 kcal mol^–1^ less stable than the conformer needed to form the triple helix, **L-3** (Fig. 4C). Since d-amino acids have been shown to destabilize the triple helix,^5^,^18^ azPro's energetic preference for the l-configuration when arranged in the azPOG triplet should help facilitate the stable triple helical assembly of **1**. While the system shows a conformational preference for **L-3**, the inversion barrier indicates that this does not preclude **D-3** from being populated. This contrasts with a stereostatic Pro equivalent in which only both enantiomers can exist after racemization. This distinction could facilitate the use of entropic or enthalpic external factors to control the stereodynamics of azPro in peptides.^19^

In this study, crystallographic data verifies that the added N atom in azPro adopts a pyramidalized conformation and that azPro is a near perfect mimic for Pro in this CMP. Furthermore, DFT calculations indicate that the conformation of azPro in this CMP is energetically favorable and that azPro is not simply forced into this conformation by the overall helical structure of the peptide. Elucidating these previously uncharacterized structural properties of azPro in collagen will enable and inform future studies of the biophysical properties of aza-amino acids in biologically relevant molecules. In addition, these findings highlight the teleological importance of stereochemical preorganization on self-assembly in collagen. Although azPro can structurally mimic l-proline in collagen, because of the slower rate of triple helical self-assembly, configurationally labile amino acids would dramatically alter collagen's evolutionary timescales. Using azPro as a stereodynamic probe can reveal fundamental insight into the interplay of structural forces and folding processes in biomolecular systems.

## Experimental procedures

A detailed explanation of all procedures for synthesis, purification, characterization, crystallography, and computational analysis can be found in the ESI.† Crystallographic data for CMPs **1** and **2** including atomic coordinates and structure factors are deposited in the Protein Data Bank (PDB) with accession codes ; 6M80 and ; 5K86, respectively.

## Conflicts of interest

There are no conflicts to declare.

## Supplementary Material

Supplementary information

Supplementary information
