# Supplementary material for: Aza-proline effectively mimics l-proline stereochemistry in triple helical collagen
Source: Chem Sci. 2019 Jun 21;10(29):6979–83. doi: 10.1039/c9sc02211b (PMC6761869; doi:10.1039/c9sc02211b)
Supplement: Supplementary file 2 [file SC-010-C9SC02211B-s002.pdf]

Electronic Supplementary Material (ESI) for Chemical Science.
This journal is © The Royal Society of Chemistry 2019

Aza-Proline Effectively Mimics L-Proline Stereochemistry in Triple Helical Collagen

Alexander J. Kasznel,[a,b] Trevor Harris,[a] Nicholas J. Porter,[a] Yitao Zhang[a] and David M. Chenoweth*[a]

[a] *Department of Chemistry, University of Pennsylvania, 231 S. 34th St., Philadelphia, PA 19104-6323, USA. Email: dcheno@sas.upenn.edu*
[b] *Department of Bioengineering, University of Pennsylvania, 210 S. 33rd St., Philadelphia, PA 19104-6323, USA*

Table of Contents

General Information 3-4
Characterization of Extent of Pyramidalization in Molecules Containing *N*-Amidourea Moieties 5-8
Figure S1. Extent of pyramidalization in *N*-amidourea-containing structures 5
Table S1. Full identification and pyramidalization details of *N*-amidourea-containing structures 6
Figure S2. Classification of *N*-amidourea-containing structures 7-8
Figure S3. Average *d* values of N_y in *N*-amidoureas 8

Table S2. Twisted nature of amide in azPro residue in each strand of collagen triple helix 9

Pyramidalization Parameters and Main-Chain Dihedrals of azPro and Pro Residues in CMPs **1** and **2** 10
Table S3. Pyramidalization parameters and main-chain dihedrals of azPro residue in each strand of CMP **1** 10
Table S4. Pyramidalization parameters and main-chain dihedrals of Pro residue in each strand of CMP **2** 10

Characterization of Extent of Pyramidalization in Molecules Containing Urea or Hydrazide Moieties 11
Figure S4. Range of *d* values for ureas and hydrazides 11

N-CO Distances of Urea Moieties 12
Figure S5. Range of N-CO distances in urea moieties 12
Table S5. N-CO distances of urea moiety in CMP **1** 12

RMSD Analysis of CMP **1** and CMP **2** 13

Synthesis and Purification of CMP **1** 14-15
Scheme S1. Overall scheme for solid-phase synthesis of CMP **1** 14
Synthesis and Purification of Fmoc-GlyAzProHyp(tBu)-OH Synthon 16-17
Scheme S2. Overall scheme for synthesis of Fmoc-GlyAzProHyp(tBu)-OH synthon 16
NMR Spectra of **4** and **7** 18-19
Figure S6. ^{1}H and ^{13}C NMR spectra of **4** in DMSO-d_6 18
Figure S7. ^{1}H and ^{13}C NMR spectra of **7** in DMSO-d_6 19

HPLC Trace and MALDI-TOF Mass Spectrum of CMP **1** 20
Crystallization 21
Crystal Structure Refinement 21
Table S6. Data collection and refinement statistics 21

Calculated Geometries 22-26
References 27
Author Contributions 27

Experimental Procedures

General Information

Throughout this document, the following abbreviations are used:

ACN	Acetonitrile
AcOH	Acetic acid
CD	Circular dichroism
CDI	1-1'-Carbonyldiimidazole
CHCA	α-Cyano-4-hydroxycinnamic acid
CMP	Collagen model peptide
DBU	1,8-Diazabicyclo[5.4.0]undec-7-ene
DCM	Dichloromethane
DFT	Density functional theory
DIEA	*N,N*-Diisopropylethylamine
DMF	*N,N*-Dimethylformamide
ESI	Electrospray ionization
EtOAc	Ethyl acetate
EtOH	Ethanol
EDT	Ethane-1,2-dithiol
Fmoc	9-Fluoroenylmethyloxycarbonyl
HATU	1-[Bis(dimethylamino)methylene]-1H-1,2,3-triazolo[4,5-b]pyridinium 3-oxid hexafluorophosphate
Hex	Hexanes
HOBt	Hydroxybenzotriazole
HPLC	High-performance liquid chromatography
HRMS	High-resolution mass spectrometry
IBCF	Isobutyl chloroformate
MALDI-TOF MS	Matrix-assisted laser desorption/ionization time-of-flight mass spectrometry
MeOH	Methanol
NMM	*N*-Methylmorpholine
NMR	Nuclear magnetic resonance
Pbf	2,2,4,6,7-Pentamethyldihydrobenzofuran-5-sulfonyl
PBS	Phosphate-buffered saline
SPPS	Solid-phase peptide synthesis
tBu	*tert*-Butyl
TFA	Trifluoroacetic acid
TFE	2,2,2-Trifluoroethanol
THF	Tetrahydrofuran
TLC	Thin-layer chromatography
TS	Transition state
XRD	X-ray diffraction

Reagents:

All commercially available solvents and reagents were used as received. Rink Amide resin was purchased from Novabiochem. HATU was purchased from Oakwood Chemical. DIEA and diethyl ether were purchased from Sigma-Aldrich. Fmoc-Arg(Pbf)-OH and Fmoc-L-Pro-OH were purchased from Chem-Impex International. Fmoc-Gly-OH and 2-chlorotrityl chloride resin were purchased from Advanced ChemTech. DBU, Phenol, and TFA were purchased from Acros Organics. EDT was purchased from Aldrich Chemical Company. Lithium sulfate monohydrate ($Li_2SO_4 \cdot H_2O$) was purchased from Sigma Life Science. PEG4000 was purchased from Hampton Research. All other commercially available solvents and reagents were purchased from Fisher Scientific or Sigma-Aldrich. Fmoc-PO(tBu)G-OH and Fmoc-GazPO(tBu)-OH were prepared in-house using previously published techniques (synthesis of Fmoc-GazPO(tBu)-OH outlined on pages 16-17).[1-3] Fmoc-hydrazine was prepared in-house using a protocol established by Carpino and Han[4] and also utilized previously in our lab.[2]

Instrumentation:

HPLC was performed using a JASCO PU-2080 Plus Intelligent HPLC Pump and Phenomenex columns (Semi-prep: Luna C18(2), 250 x 10 mm, 5 µm particle size, 100 Å pore size; Analytical: Luna Omega PS C18, 250 x 4.6 mm, 5 µm particle size, 100 Å pore size). MALDI-TOF MS was performed using a Bruker MALDI-TOF Ultraflex III Mass Spectrometer. CHCA was used as the matrix for all MALDI-TOF MS measurements. Peptide was lyophilized using a Labconco FreeZone Plus 12 Liter Cascade Console Freeze Dry System. CD measurements were performed using a JASCO J-1500 Circular Dichroism Spectrometer. UV-vis measurements were performed using a JASCO V-650 UV-vis Spectrophotometer. XRD was performed using the 24-ID-C undulator beamline operated by the Northeastern Collaborative Access Team (NE-CAT) at the Advanced Photon Source (APS) (Argonne National Laboratory, Argonne, IL). Proton nuclear magnetic resonance spectroscopy (^1H NMR) and carbon nuclear magnetic resonance spectroscopy (^{13}C NMR) spectra were recorded on a Bruker UNI 500 ^1H NMR. High-resolution mass spectra were obtained at the University of Pennsylvania's Mass Spectrometry Service Center on a Micromass AutoSpec electrospray/chemical ionization spectrometer. Flash chromatography was performed using a Teledyne ISCO CombiFlash Rf chromatography system.

Computational Details:

All DFT calculations were carried with the Gaussian 09 software package[5] using the M06-2X[6, 7] DFT functional with the 6-31+G(d,p) basis set for all atoms. The implicit SMD[8] solvation model was used to simulate the effects of water throughout the calculated structures. Frequency calculations were carried out for all structures to confirm them as either a minimum or a TS. Three-dimensional structures were produced with UCSF Chimera.[9]

RMSD values reported in the manuscript and SI were calculated using UCSF Chimera. For the RMSD reported in Figure 3 comparing the full backbone and sidechain conformations of CMPs **1** & **2**, models were aligned using the MatchMaker tool and solvent molecules and hydrogen atoms were removed. The two peptides (468 atom pairs) were then selected and compared using the **rmsd sel** command. For Figure 4A and for RMSD Analysis of CMPs **1** & **2** in the SI, the RMSD values were calculated in UCSF Chimera using the **match** command. The **match** command performs least-squares fit root-mean-square deviations of specified atoms, moving the first set of atoms (by default, the entire models containing them) onto the second.

Results and Discussion

Characterization of Extent of Pyramidalization in Molecules Containing *N*-Amidourea Moieties

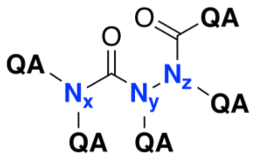

QA = Query Atom [C or H]

Category	Average *d* (Å)		
	N_x	N_y	N_z
1	0.059	0.021	0.005
2	0.056	0.075	0.047
3	0.073	0.130	0.090
4	0.122	0.173	0.019
5	0.003	0.211	0.006
6	0.073	0.296	0.126
7	0.034	0.362	0.159

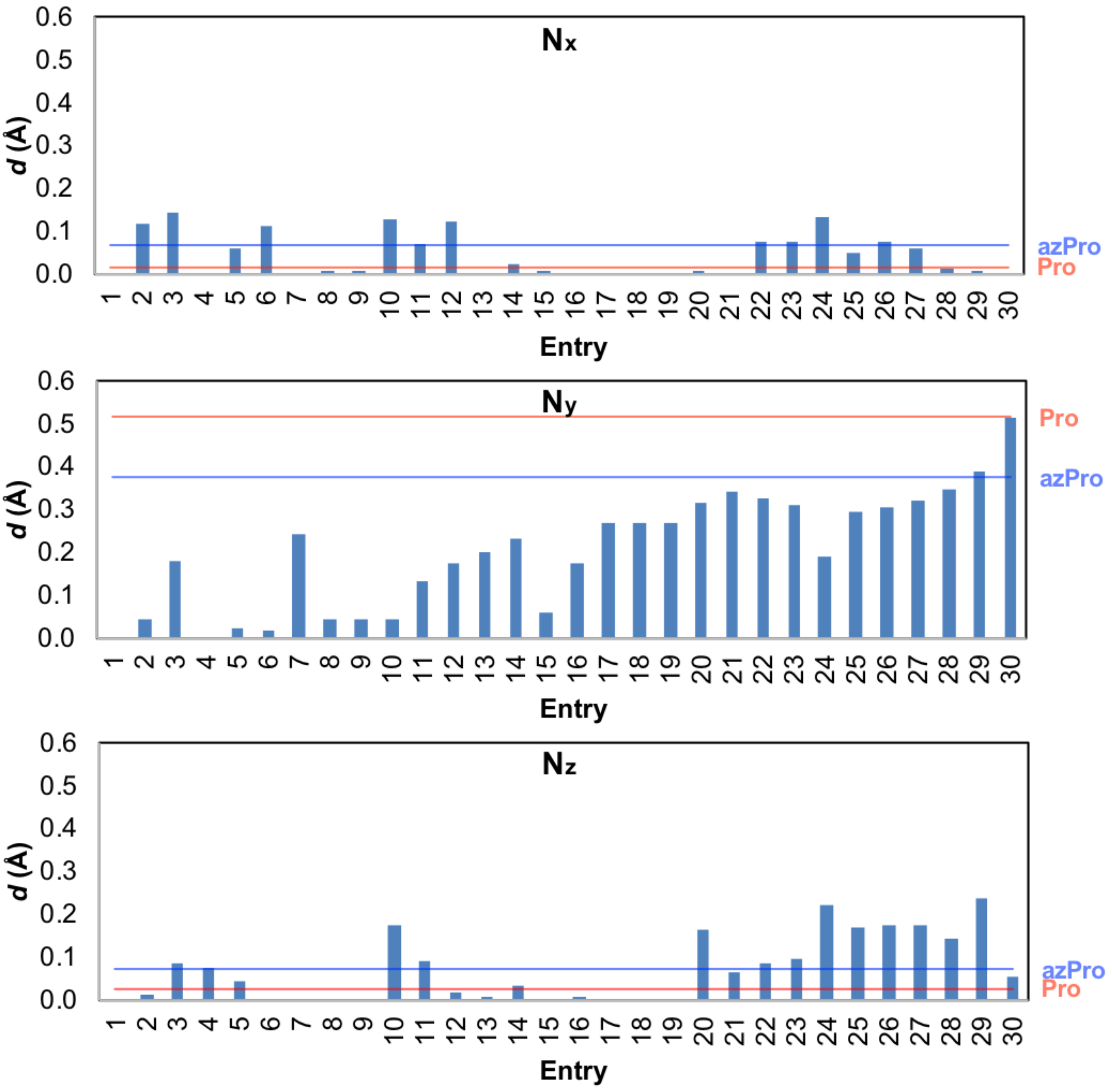

Figure S1. Extent of pyramidalization in *N*-amidourea-containing structures. A substructure search of the Cambridge Structural Database (CSD)[10] was performed using the search structure shown. The search results were then categorized according to the extent of substitution of the N_x, N_y, and N_z atoms in each structure (see Figure S2). In the table of average *d* values, categories containing structures in which N_y is a member of a ring are highlighted in blue. In each plot, the average *d* values for Pro7 and azPro7 in PDB 5K86 and 6M80, respectively, are shown for comparison. Parameter *d* was calculated for N_x, N_y, and N_z in each structure as described in Tables S3-S4 below using Mercury 4.0.0 (Build 224311)[11, 12] (for structures from the CSD) or UCSF Chimera[9] (for CMPs).

Table S1. Full identification and pyramidalization details of *N*-amidourea-containing structures. Structure MORHIK was omitted from search results because its CSD entry did not contain any 3D coordinates to perform the necessary measurements. Black cell = Coordinates missing from CSD entry; could not complete measurement; [a] For CSD structures containing multiple monomers of the constituent molecule, a single monomer was used as a representative sample for statistical analysis; [b] For CSD structures containing multiple matches for the substructure search terms shown in Figure S1 above (i.e. multiple *N*-amidourea moieties), each instance of the substructure motif was treated as a separate entry.

Entry	CCDC ID	CSD Deposition #	Crystal ID	*d* (Å)			Category
				N_x	N_y	N_z	
1	FUZQEX	769209	sx2752	0.000	0.000	0.000	1
2	UGIWOX	687188	I	0.118	0.042	0.010	1
3	JORZEV	1189128	JORZEV	0.142	0.180	0.084	2
4	KEKYUV	602195	Compound4c	0.000	0.000	0.076	2
5	LERTAD	134079	LERTAD	0.060	0.025	0.044	2
6	RAYHEG	837003	Greg1588f	0.114	0.017	0.000	2
7	RUNDIO	725824	aza3	0.000	0.244	0.000	2
8	TUPMAS	1277042	TUPMAS	0.001	0.042	0.000	2
9	TUPMAS01	108192	fix	0.001	0.042	0.000	2
10	XIQGUZ	1296757	XIQGUZ	0.127	0.046	0.175	2
11	RAYHAC	837002	Greg1405f	0.073	0.130	0.090	3
12	ZEXXIL	896854	2186f	0.122	0.173	0.019	4
13	QAZZID[b]	1533841	mam16_100k_2	0.000	0.202	0.009	5
14				0.023	0.232	0.033	
15	QAZZUP[a],[b]	1537325	mam36	0.001	0.060	0.000	5
16				0.000	0.175	0.001	
17	TIXFIQ[b]	683660	c3isoallyl	0.000	0.270	0.000	5
18				0.000	0.270	0.000	
19				0.000	0.270	0.000	
20	XITLET	962966	mont06s	0.001	0.316	0.162	6
21	NUXWOS	1225054	NUXWOS		0.339	0.065	6
22	OBEZAW[a]	127839	AzPip-Ala	0.078	0.326	0.086	6
23	OBEZEA	127838	Ala-AzPip	0.078	0.309	0.095	6
24	LAGLIO	1202742	LAGLIO	0.134	0.189	0.222	6
25	PIMYIV	941199	compound10d	0.049	0.296	0.169	7
26	QAHSUQ[a]	910352	compound-3c	0.075	0.306	0.176	7
27	QAHTAX	910351	compound-3a	0.060	0.321	0.175	7
28	QAHTEB	910129	mo438	0.011	0.347	0.144	7
29	PILDAP	1233742	PILDAP	0.010	0.389	0.239	7
30	MISGIG	917328	compound9	0.000	0.515	0.052	7

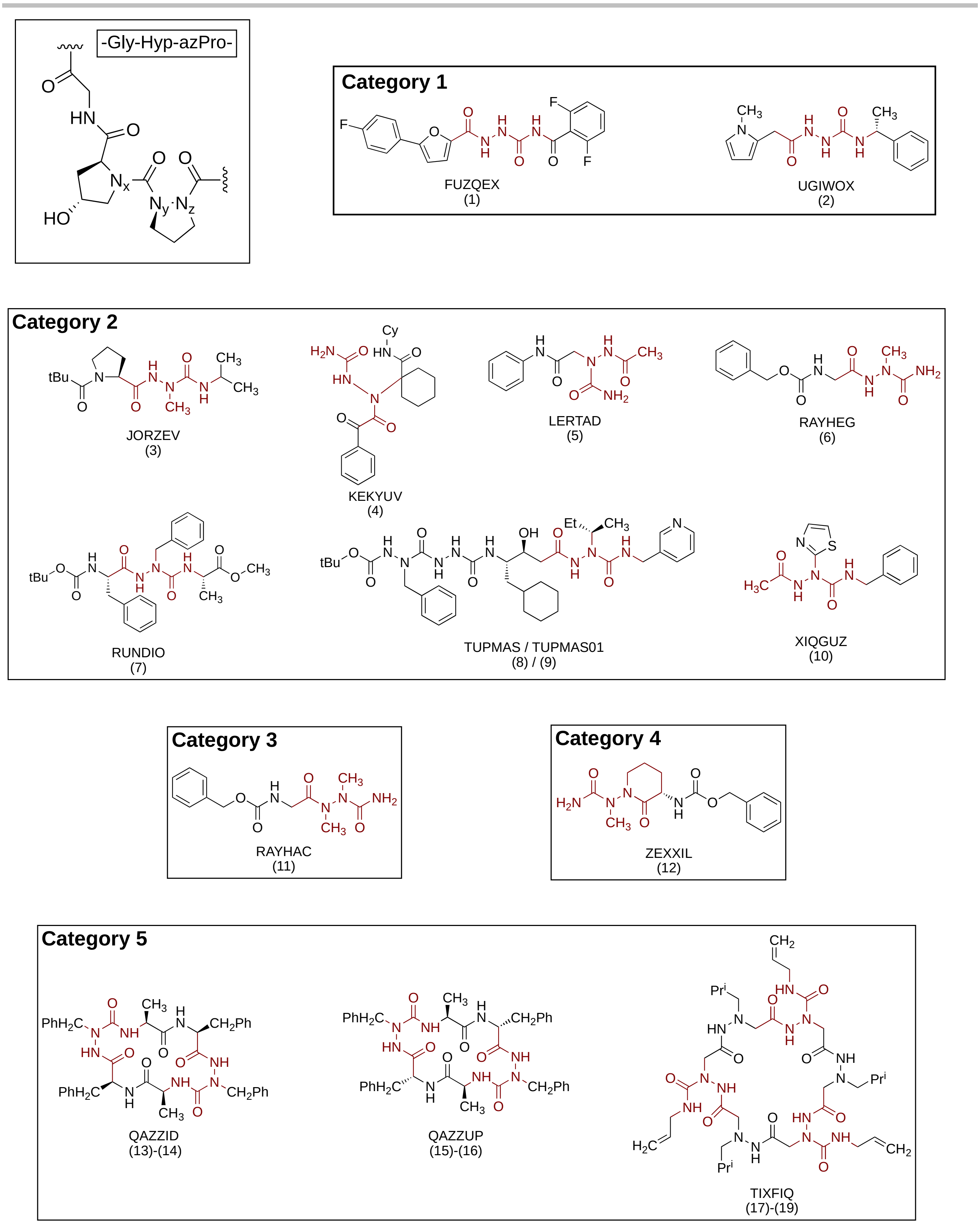

Figure S2. Classification of *N*-amidourea-containing structures. Structures were sorted into categories according to the extent of substitution of the N_x, N_y, and N_z atoms in each, as well as the local environment of the N_y atom (e.g. member of a chain [Categories 1-4], macrocycle [Category 5], or ring [Categories 6-7]). The Gly-Hyp-azPro fragment found in CMP **1** is illustrated for comparison. 2D structures of search results are shown with their respective *N*-amidourea fragments highlighted.

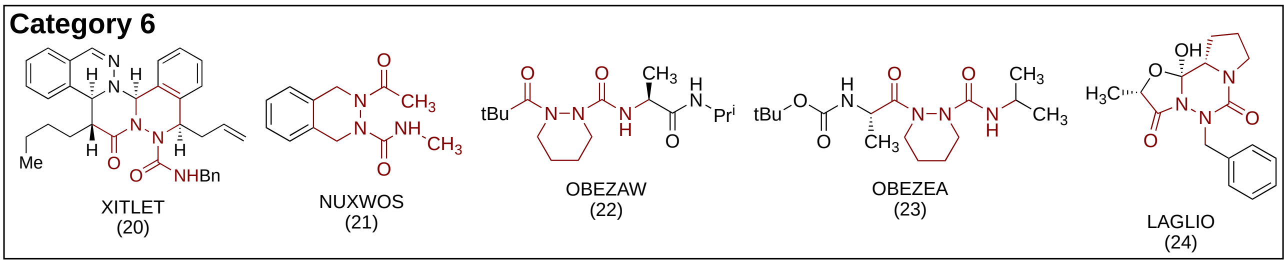

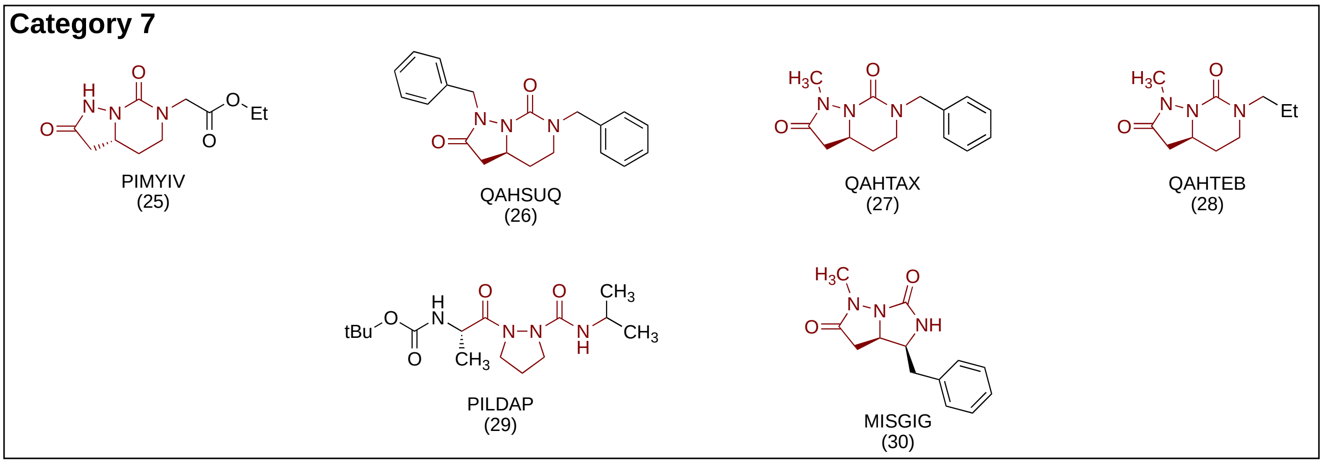

Figure S2, cont'd. Classification of *N*-amidourea-containing structures. Structures were sorted into categories according to the extent of substitution of the N_x, N_y, and N_z atoms in each, as well as the local environment of the N_y atom (e.g. member of a chain [Categories 1-4], macrocycle [Category 5], or ring [Categories 6-7]). The Gly-Hyp-azPro fragment found in CMP **1** is illustrated for comparison. 2D structures of search results are shown with their respective *N*-amidourea fragments highlighted.

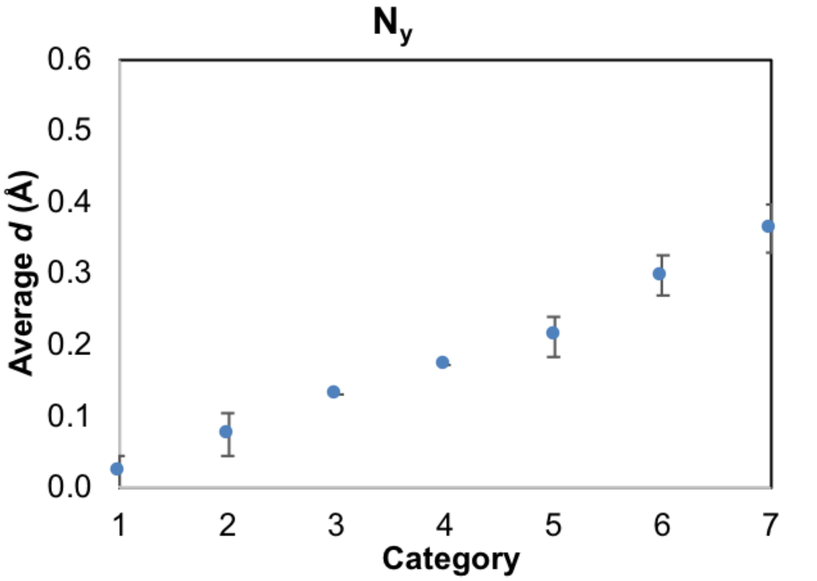

Figure S3. Average d values of N_y in *N*-amidoureas. The average *d* values for the N_y atoms in each category of *N*-amidoureas are shown. In the CMP crystal structure presented in this publication (CMP **1**; PDB 6M80), N_y corresponds to the added backbone N atom in azPro. As the degree of substitution of N_y increases, the *d* value (i.e. extent of pyramidalization) increases. The *d* values of N_y are greatest in molecules in which N_y is a member of a macrocycle/ring (Categories 5-7) (Error bars = SEM).

Table S2. Twisted nature of amide in azPro residue in each strand of collagen triple helix. Data taken from PDB 6M80 for azPro7. The twist angle (τ)[13, 14] describes the magnitude of rotation around the N–CO amide bond. A twist angle (τ) of 0° corresponds to a planar amide and a twist angle of 90° corresponds to a fully orthogonal twisted amide.

(1)	τ (°)
**Strand C**	**28.7**
**Strand D**	**34.1**
**Strand E**	**34.0**

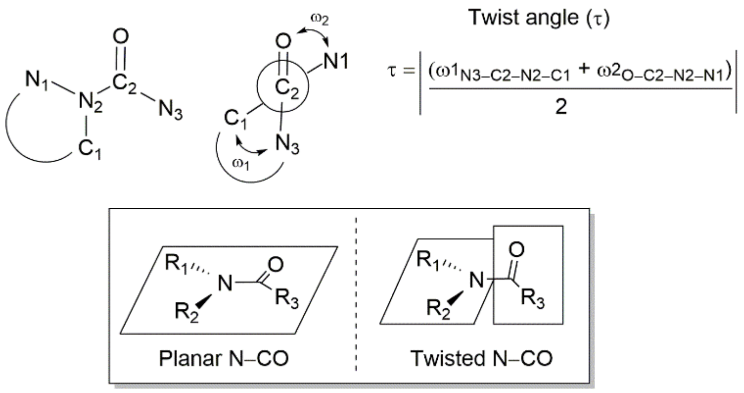

Pyramidalization Parameters and Main-Chain Dihedrals of azPro and Pro Residues in CMPs (1) and (2)

Table S3. Pyramidalization parameters and main-chain dihedrals of azPro residue in each strand of CMP (1). Data taken from PDB 6M80 for azPro7. Φ (CNNC) and Ψ (NCNN) main-chain dihedrals are in degrees. δ is a measurement of pyramidal character (in degrees) and is defined as δ = S – 360°, where S is the sum of the valence angles around the atom of interest (αN). If δ = 0, then the site is fully planar. As δ becomes more negative, the magnitude of pyramidalization increases (sp^3 character).[15] The hinge angle (α) is a measurement of pyramidal character (in degrees) and is defined as the angle from the plane of the αN and the two adjacent atoms with respect to the carbonyl carbon atom. The hinge angle (α) is between 180° (pure sp^2) and 125° (pure sp^3).[13, 14] Another measurement of pyramidalization (*d*) is defined using a triangular pyramid with the αN at the apex and substituent atoms positioned at the remaining vertices of the base. The distance from the apex normal to the base plane is measured as the *d* value, in Å.

(**1**)	Φ (°)	Ψ (°)	δ (°)	α (°)	*d* (Å)
Strand C	-88.9	173.9	-20.4	137.7	0.357
Strand D	-87.4	171.0	-24.4	133.9	0.389
Strand E	-81.0	167.4	-23.2	134.9	0.377
Std. Dev.	4.2	3.3	2.1	2.0	0.016

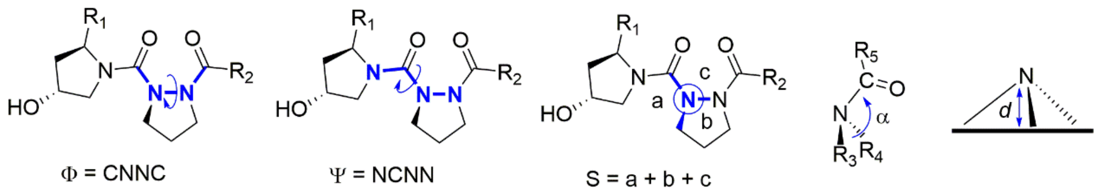

Table S4. Pyramidalization parameters and main-chain dihedrals of Pro residue in each strand of CMP (2). Data taken from PDB 5K86 for Pro7. Φ (CCNC) and Ψ (NCCN) main-chain dihedrals are in degrees. δ is a measurement of pyramidal character (in degrees) and is defined as δ = S – 360°, where S is the sum of the valence angles around the atom of interest (αC). If δ = 0, then the site is fully planar. As δ becomes more negative, the magnitude of pyramidalization increases (sp^3 character).[15] The hinge angle (α) is a measurement of pyramidal character (in degrees) and is defined as the angle from the plane of the αC and the two adjacent atoms with respect to the carbonyl carbon atom. The hinge angle (α) is between 180° (pure sp^2) and 125° (pure sp^3).[13, 14] Another measurement of pyramidalization (*d*) is defined using a triangular pyramid with the αC at the apex and substituent atoms positioned at the remaining vertices of the base. The distance from the apex normal to the base plane is measured as the *d* value, in Å.

(**2**)	Φ (°)	Ψ (°)	δ (°)	α (°)	*d* (Å)
Strand A	-81.3	172.8	-34.2	125.8	0.520
Strand B	-78.2	164.4	-35.0	125.2	0.525
Strand C	-78.2	168.3	-32.3	127.5	0.502
Std. Dev.	1.8	4.2	1.4	1.2	0.012

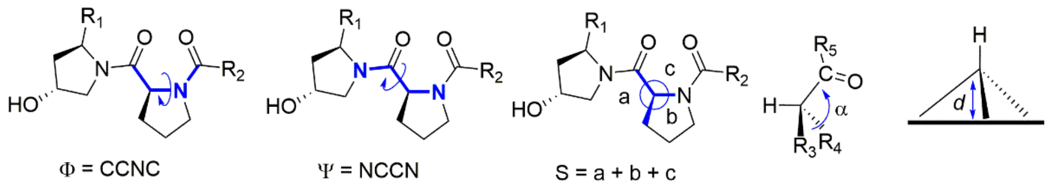

Characterization of Extent of Pyramidalization in Molecules Containing Urea or Hydrazide Moieties

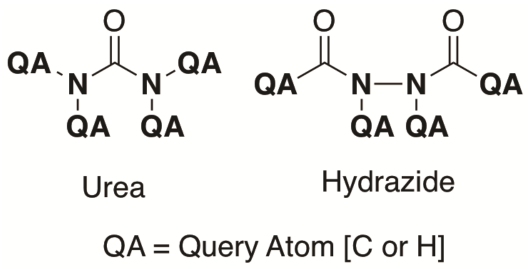

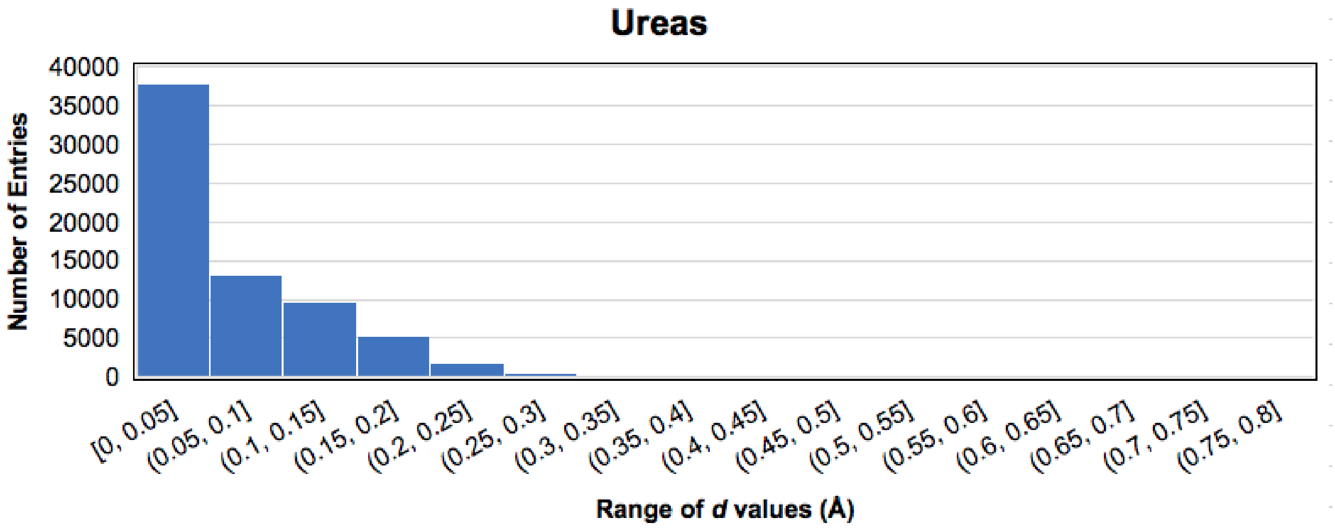

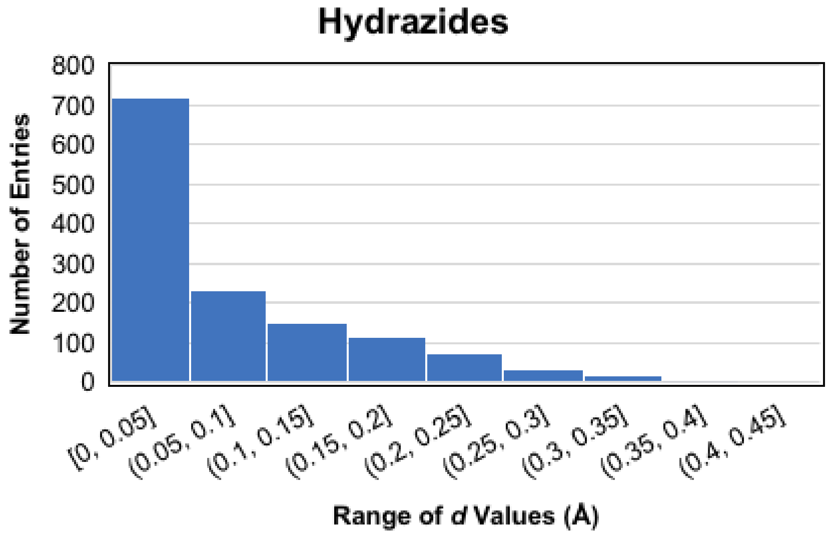

Figure S4. Range of *d* values for ureas and hydrazides. Substructure searches of the Cambridge Structural Database (CSD)[10] were performed using the search structures shown (Ureas: n = 12317; hydrazides: n = 498). For each search, parameter *d* was defined for each backbone N atom as described in Tables S3-S4 above. Each *d* value was treated as a separate entry, and results were summarized in histograms. Searches were performed using ConQuest (Version 2.0.0 [Build 224359]) (CSD version 5.40 [November 2018]).[16]

N-CO Distances of Urea Moieties

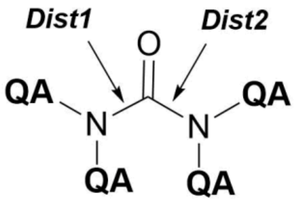

QA = Query Atom [C or H]

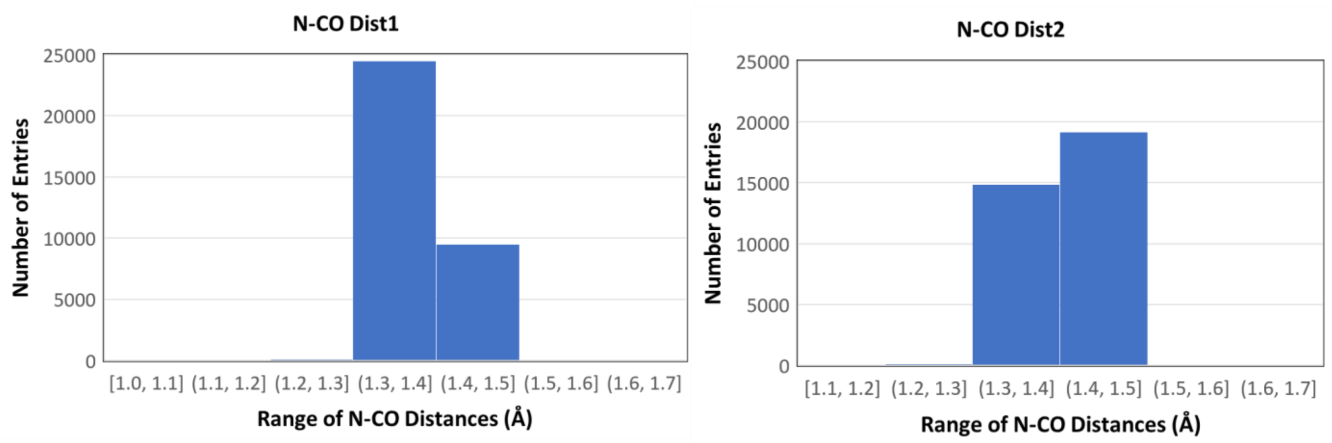

Figure S5. Range of N-CO distances in urea moieties. Substructure searches of the Cambridge Structural Database (CSD)[10] were performed using the search structures shown (n = 12317). Each urea moiety in search structures was treated as a separate entry, and results were summarized in histograms. Searches were performed using ConQuest (Version 2.0.2 [Build 246535]) (CSD version 5.40 [November 2018]).[16]

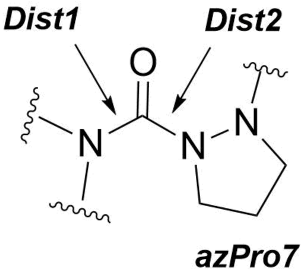

	Dist1	Dist2
Strand 1	1.34 Å	1.42 Å
Strand 2	1.40 Å	1.48 Å
Strand 3	1.36 Å	1.44 Å

Table S5. N-CO distances of urea moiety in CMP (1). Data taken from PDB 6M80, illustrating divergent N-CO distances.

RMSD Analysis of CMP (1) and CMP (2)

Dipeptide Comparison

-(PDB 6M80/Chain C) **azPro7-Hyp8** with (PDB 5K86/Chain A) **Pro7-Hyp8**

15 atom pairs: 0.121 Å

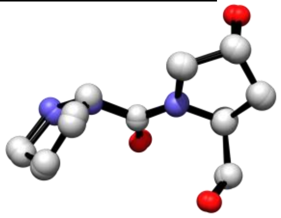

-(PDB 6M80/Chain D) **azPro7-Hyp8** with (PDB 5K86/Chain B) **Pro7-Hyp8**

15 atom pairs: 0.155 Å

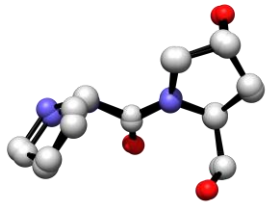

-(PDB 6M80/Chain E) **azPro7-Hyp8** with (PDB 5K86/Chain C) **Pro7-Hyp8**

15 atom pairs: 0.099 Å

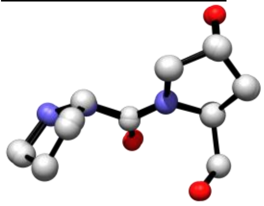

AzPro7 and Pro7 Comparison

-(PDB 6M80/Chain C) **azPro7** with (PDB 5K86/Chain A) **Pro7**

7 atom pairs: 0.124 Å

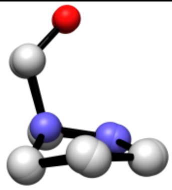

-(PDB 6M80/Chain D) **azPro7** with (PDB 5K86/Chain B) **Pro7**

7 atom pairs: 0.134 Å

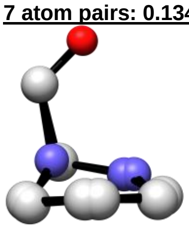

-(PDB 6M80/Chain E) **azPro7** with (PDB 5K86/Chain C) **Pro7**

7 atom pairs: 0.109 Å

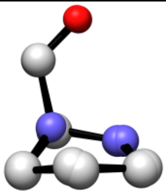

Synthesis and Purification of CMP (1)

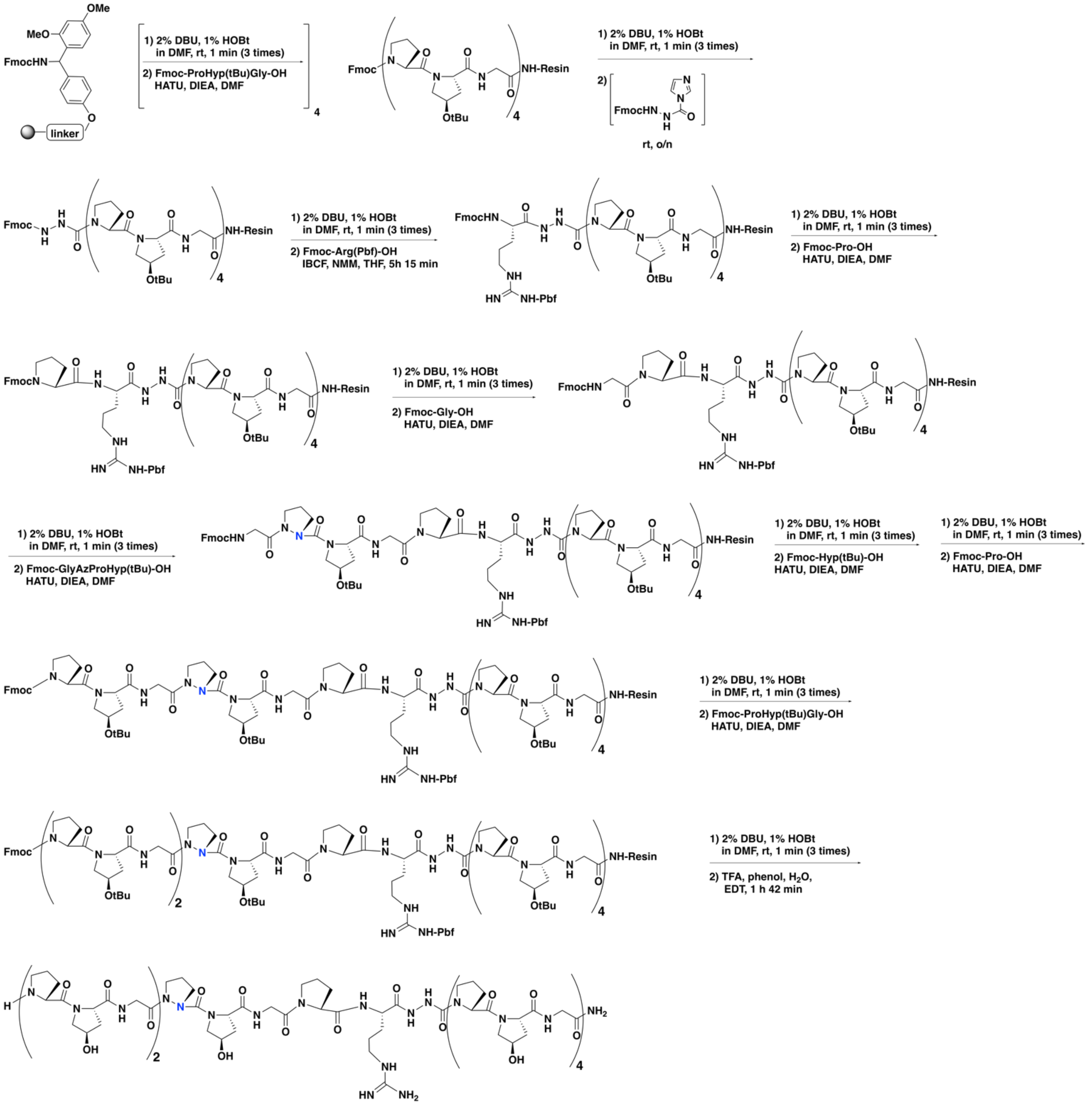

H-(Pro-Hyp-Gly)$_2$-azPro-Hyp-Gly-Pro-Arg-azGly-(Pro-Hyp-Gly)$_4$-NH$_2$

1

Scheme S1. Overall scheme for solid-phase synthesis of CMP (1).

Synthesis

Peptide **1** was synthesized using manual SPPS on Rink Amide resin (0.54 mmol/g) using Fmoc as the primary protecting group. When it was necessary to stop the synthesis after completing a coupling, the post-coupling wash step was modified such that the resin was washed with DMF (3x) and DCM (3x); following the Arg coupling, additional washes with EtOH were also performed (see step 4 below). When beginning from a dry resin at any point during the synthesis, the resin was swelled for at least 30 min in DMF prior to initial deprotection. The synthesis of peptide **2** was reported previously.[17]

1. Resin preparation

37 mg (0.02 mmol) Rink Amide resin was added to a SPPS vessel. The resin was swelled by stirring in DMF for 30 min. A deprotecting stock solution was prepared by combining 20 mL DMF, 200 mg HOBt, and 0.4 mL DBU (1% HOBt (w/v), 2% DBU (v/v) in DMF). The Fmoc protecting group was removed from the resin by mixing with 1 mL of this deprotecting solution and then draining the solution (3 x 1 min).

2. Fmoc-ProHyp(tBu)Gly-OH synthon coupling
Following initial deprotection, the resin was washed with DMF (6x). A stock solution of HATU was prepared by dissolving 690 mg (1.81 mmol) HATU in 20 mL DMF. This solution was then used to prepare coupling solutions as noted. A coupling solution of 34 mg (0.06 mmol, 3 eq.) Fmoc-ProHyp(tBu)Gly-OH, 0.67 mL (0.06 mmol, 3 eq.) HATU in DMF, and 20 µL DIEA (0.12 mmol, 6 eq.) was prepared in a 4-mL vial and allowed to activate for ~10 min at room temperature. This solution was then added to the resin in the SPPS vessel and stirred for 40-70 min. The coupling solution was drained from the vessel and the resin was washed with DMF (6x). These steps were repeated 4 times to couple a total of 4 ProHyp(tBu)Gly trimers onto the resin.

3. Aza-glycine (azGly) coupling
The Fmoc protecting group was removed as described above, and the resin was washed with DMF (6x). A coupling solution of 10 mg (0.06 mmol, 3 eq.) CDI, 0.4 mL DMF, and 15 mg (0.06 mmol, 3 eq.) Fmoc-hydrazine (Fmoc-NH-NH_2) was mixed in a vial and allowed to activate for 5-10 min at room temperature. The coupling solution was then added to the resin and stirred overnight. The following day, a fresh coupling solution was prepared and allowed to activate as described above. The original coupling solution was drained from the vessel and the fresh solution was added to the resin and stirred for an additional 4 h. This second coupling solution was drained from the vessel and the resin was washed with DMF (6x).

4. Arginine (Arg) coupling
The Fmoc protecting group was removed as described above, and the resin was washed with DMF (5x) followed by THF (2x). A coupling solution of 39 mg (0.06 mmol, 3 eq.) Fmoc-Arg(Pbf)-OH, 8 µL IBCF (0.06 mmol, 3 eq.), 13 µL NMM (0.12 mmol, 6 eq.), and 0.65 mL THF was prepared and allowed to activate for ~10 min at room temperature. This solution was then added to the resin and stirred for 5 h 15 min. The coupling solution was drained and the resin was washed with EtOH (2x), DMF (3x), and DCM (3x).

5. Proline (Pro) coupling
The Fmoc protecting group was removed as described above, and the resin was washed with DMF (6x). A coupling solution of 20.2 mg (0.06 mmol, 3 eq.) Fmoc-L-Pro-OH, 0.67 mL (0.06 mmol, 3 eq.) HATU, and 20 µL DIEA (0.12 mmol, 6 eq.) was prepared and allowed to activate for ~10 min at room temperature. This solution was then added to the resin and stirred for 45 min. The coupling solution was drained from the vessel and the resin was washed with DMF (6x).

6. Glycine (Gly) coupling
The Fmoc protecting group was removed as described above, and the resin was washed with DMF (6x). A coupling solution of 18 mg (0.06 mmol, 3 eq.) Fmoc-Gly-OH, 0.67 mL (0.06 mmol, 3 eq.) HATU, and 20 µL DIEA (0.12 mmol, 6 eq.) was prepared and allowed to activate for ~10 min at room temperature. This solution was then added to the resin and stirred for 1 h 30 min. The coupling solution was drained from the vessel and the resin was washed with DMF (6x).

7. Fmoc-GlyAzProHyp(tBu)-OH synthon coupling
The Fmoc protecting group was removed as described above, and the resin was washed with DMF (6x). A coupling solution of 34.0 mg (0.06 mmol, 3 eq.) Fmoc-GlyAzProHyp(tBu)-OH, 0.67 mL (0.06 mmol, 3 eq.) HATU, and 20 µL DIEA (0.12 mmol, 6 eq.) was prepared in a 4-mL vial and allowed to activate for ~10 min at room temperature. This solution was then added to the resin and stirred for 50 min. The coupling solution was drained from the vessel and the resin was washed with DMF (6x).

8. Hydroxyproline [Hyp(tBu)] coupling
The Fmoc protecting group was removed as described above, and the resin was washed with DMF (6x). A coupling solution of 24.6 mg (0.06 mmol, 3 eq.) Fmoc-L-Hyp(tBu)-OH, 0.67 mL (0.06 mmol, 3 eq.) HATU, and 20 µL DIEA (0.12 mmol, 6 eq.) was prepared and allowed to activate for ~10 min at room temperature. This solution was then added to the resin and stirred for 40 min. The coupling solution was drained from the vessel and the resin was washed with DMF (6x).

9. Proline (Pro) coupling
The Fmoc protecting group was removed as described above, and the resin was washed with DMF (6x). A coupling solution of 20.2 mg (0.06 mmol, 3 eq.) Fmoc-L-Pro-OH, 0.67 mL (0.06 mmol, 3 eq.) HATU, and 20 µL DIEA (0.12 mmol, 6 eq.) was prepared and allowed to activate for ~10 min at room temperature. This solution was then added to the resin and stirred for 45 min. The coupling solution was drained from the vessel and the resin was washed with DMF (6x).

10. Fmoc-ProHyp(tBu)Gly-OH synthon coupling
The Fmoc protecting group was removed as described above, and the resin was washed with DMF (6x). A coupling solution of 34.0 mg (0.06 mmol, 3 eq.) Fmoc-ProHyp(tBu)Gly-OH, 0.67 mL (0.06 mmol, 3 eq.) HATU, and 20 µL DIEA (0.12 mmol, 6 eq.) was prepared in a 4-mL vial and allowed to activate for ~10 min at room temperature. This solution was then added to the resin and stirred for 1 h. The coupling solution was drained from the vessel and the resin was washed with DMF (6x).

11. Final deprotection and cleavage
The Fmoc protecting group was removed as described above, and the resin was washed thoroughly with DMF (6x). The completed peptide was then cleaved from the resin by mixing with a cleavage cocktail of TFA, phenol, H_2O, and EDT in an 87:5:5:3 (v/v) ratio for 1 h 42 min.

Purification

Following SPPS, the peptide was precipitated in cold diethyl ether. After initial precipitation, the cleaved peptide solution was centrifuged, the supernatant was decanted, and the solid peptide was resuspended in ether. This process was repeated for a total of 3 resuspensions. After decanting the final supernatant, the solid peptide was dissolved in 18 MΩ H_2O and stored at 4 °C. The crude peptide stock was then purified using semi-preparative reversed-phase HPLC with a mobile phase gradient of ACN in H_2O (H_2O containing 0.1% TFA). The peptide solution was heated for at least ~10 min at ~70 °C to ensure that the peptide was in the single-stranded state when loaded onto column. The chromatographic fractions were analyzed by MALDI-TOF MS in positive ion mode. The fractions found to contain the desired product were pooled according to purity and lyophilized.

Synthesis and Purification of Fmoc-GlyAzProHyp(tBu)-OH Synthon

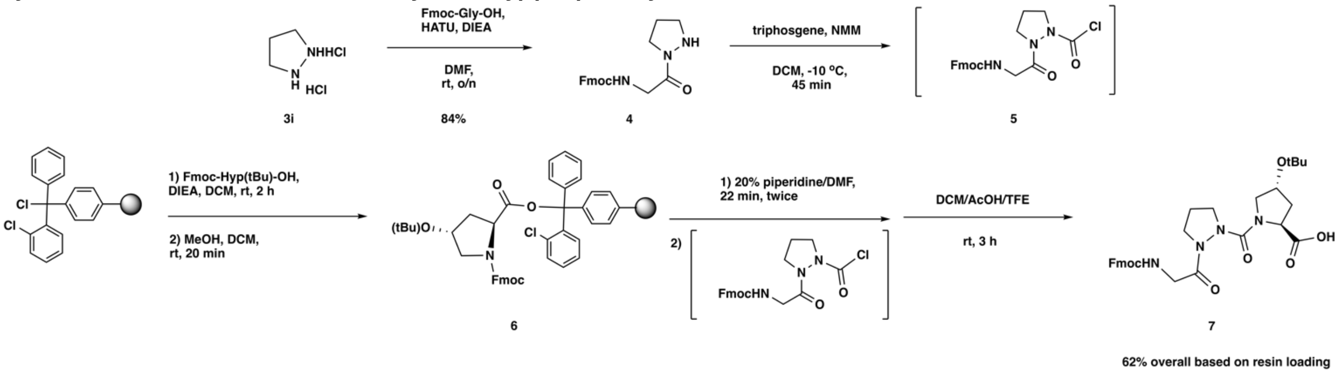

Scheme S2. Overall scheme for synthesis of Fmoc-GlyAzProHyp(tBu)-OH synthon. Compound **3i**, pyrazolidine hydrochloride, was prepared according to a procedure reported by Rabinowitz *et al.*[18]

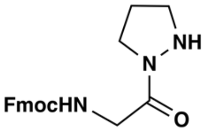

Synthesis of (9H-fluoren-9-yl)methyl (2-oxo-2-(pyrazolidin-1-yl)ethyl)carbamate (**4**)

Fmoc-Gly-OH (2.48 g, 8.33 mmol, 1.2 eq.) was dissolved in DMF (27 mL). HATU (3.17 g, 8.33 mmol, 1.2 eq.) and DIEA (5.0 mL, 28.80 mmol, 4.15 eq.) were added to the reaction mixture at 0 ºC. At this temperature, pyrazolidine hydrochloride (**3i**) (1.00 g, 6.94 mmol, 1.0 eq.) was then added to the reaction flask. The reaction mixture was stirred overnight and allowed to gradually warm to room temperature. Upon completion of the reaction, DMF was removed under vacuum. The residue was then dissolved in EtOAc, and the organic layer was sequentially washed with 5% $KHCO_3$, deionized water, and brine. The organic layer was then dried with Na_2SO_4 and concentrated. After flash chromatography (75-100% EtOAc/Hex) the product was obtained as a light flake solid (2.04 g, 84%).

TLC: R_f = 0.23 (40% EtOAc/Hex).
^{1}H NMR (500 MHz, DMSO-d6): δ 8.32 (s, 1H), 7.89 (d, J = 7.5 Hz, 2H), 7.78-7.56 (m, 2H), 7.42 (t, J = 7.5 2H), 7.36 – 7.22 (m, 3H), 5.13-5.00 (m, 1H), 4.28-4.16 (m, 3H), 3.9 (d, J = 6 Hz, 2H), 3.36-3.25 (m, 2H), 2.86-2.78(m, 2H) 1.96-1.88 (m, 2H).
^{13}C NMR (125 MHz, DMSO-d_6): δ 168.44, 156.50, 143.89, 140.71, 127.59, 127.05, 125.26, 120.07, 65.62, 47.26, 46.65, 43.86, 42.42, 26.44.
HRMS (ESI) calculated for $C_{20}H_{21}N_3O_3$ $[M+Na]^+$ 374.1481, found 374.1474.

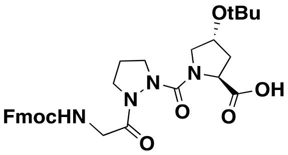

Synthesis of Fmoc-GlyAzProHyp(tBu)-OH (**7**)

For the solid-phase synthesis of Fmoc-Hyp(tBu)-loaded resin, 2-chlorotrityl chloride resin was used. To a solution of Fmoc-Hyp(tBu)-OH (5 g, 12.20 mmol) in anhydrous DCM (62 mL) in a round-bottom flask, 2-chlorotrityl chloride resin (5 g, 8.5 mmol, 1.7 mmol/g) and DIEA (2.12 mL, 12.20 mmol) were added under nitrogen. After stirring the mixture for 10 min, another portion of DIEA (3.19 mL, 18.36 mmol) was added. After stirring for 2 h, HPLC grade methanol (18 mL) was added to cap any remaining reactive trityl groups. After 20 min, the reaction mixture was filtered through filter paper, and the solid was washed with DCM (6 x 50 mL) followed by air drying. The material was further dried *in vacuo* at room temperature. The loading was measured according to a reported protocol[19] to be 0.75 mmol/g, and the total mass obtained was 5.27 g (3.94 mmol).

The Fmoc-Hyp(tBu)-loaded 2-chorotrityl chloride resin (2.67 g, 2 mmol, 1 eq.) was suspended in DMF in a SPPS vessel to swell (~40 mL, 15 min, twice). After draining the DMF used to swell the resin, the base-labile Fmoc protecting group was removed with 20% piperidine/DMF at room temperature with stirring (35 mL, 22 min, twice). The resin was washed with DMF (30 mL x 1) and DCM (30 mL x 5). After washing, 20 mL of anhydrous DCM was added to the reaction vessel, and a solution of Fmoc-GlyAzPro-Cl (**5**) in DCM, prepared as described below, was added and the reaction mixture was allowed to stir at room temperature for 5 h.

Electrophile Preparation: Converting **4** *to Intermediate* **5** *via Triphosgene*
Triphosgene (600 mg, 2 mmol, 1.0 eq.) was dissolved in anhydrous DCM (7 mL) and the solution was cooled to -10 ºC. A solution of **4** (2.04 g, 5.80 mmol, 2.9 eq.) and NMM (0.66 mL, 6.0 mmol, 3.0 eq.) in DCM (7 mL) was added to the reaction flask dropwise over 8 min. After the addition, the reaction mixture was stirred at -10 ºC for ~45 min. The material was then transferred to the SPPS vessel containing the aforementioned Fmoc-deprotected resin.

Final Cleavage & Purification

After 5 h, the solution was drained and the resin was sequentially washed with DMF (2 x 40 mL) and DCM (5 x 40 mL). The resin was dried under vacuum in the SPPS vessel at room temperature for 6 h. The dried resin was transferred into a round-bottom flask and was treated with cleavage solution consisting of DCM:AcOH:TFE (10:1:1) (131 mL:13 mL:13 mL) at room temperature for 3 h. The mixture was filtered through filter paper, and the filtrate was concentrated *in vacuo*. AcOH was removed by azeotroping with C_6H_6 (3 x 100 mL). The resulting foamy solid residue was purified by silica gel column chromatography (3.5-5% MeOH/DCM) (700 mg, 62% overall based on the determined active Fmoc-Hyp(tBu)-chlorotrityl chloride resin substitution level).

TLC: R*f* = 0.31 (5 % MeOH/DCM).
^{1}H NMR (500 MHz, DMSO-d_6): δ 7.89 (d, *J* = 7.5 Hz, 2H), 7.74-7.67 (d, *J* = 7.5 Hz, 2H), 7.42 (t, *J* = 7.5, 3H), 7.32 (t, *J* = 7.5 Hz, 2H), 4.46-3.12 (m, 13H), 2.2-1.78 (m, 4H), 1.11 (s, 9H).
^{13}C NMR (125 MHz, DMSO-d_6): δ 172.06, 168.50, 161.20, 156.51, 143.85, 140.72, 127.63, 127.08, 125.27, 120.10, 73.60, 69.75, 65.75, 58.34, 54.97, 46.69, 43.47, 41.71, 37.18, 28.10, 24.15, 21.17.
HRMS (ESI) calculated for $C_{30}H_{36}N_4O_7$ $[M+Na]^+$ 587.2482, found 587.2454.

NMR Spectra of (4) and (7)

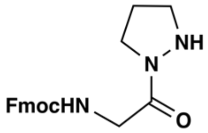

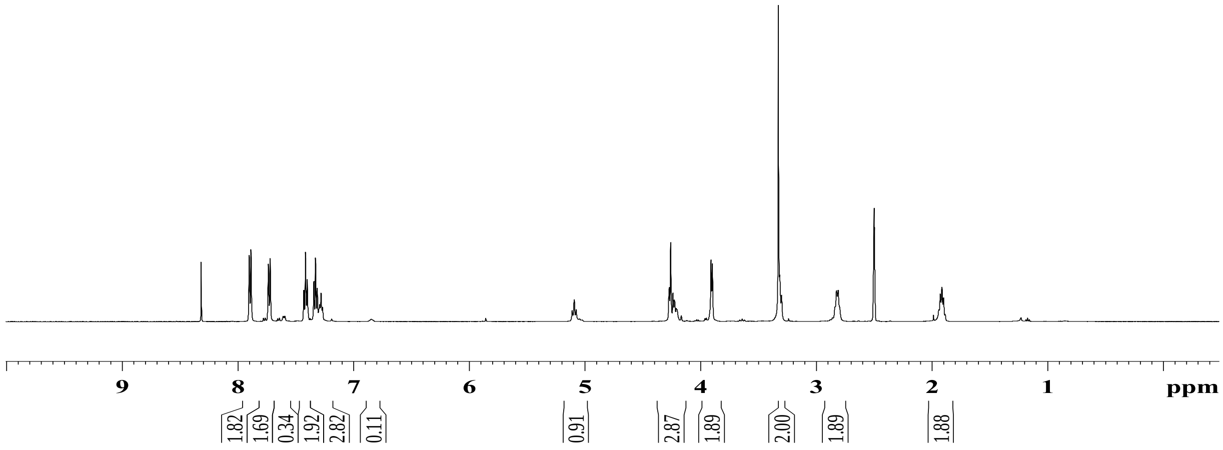

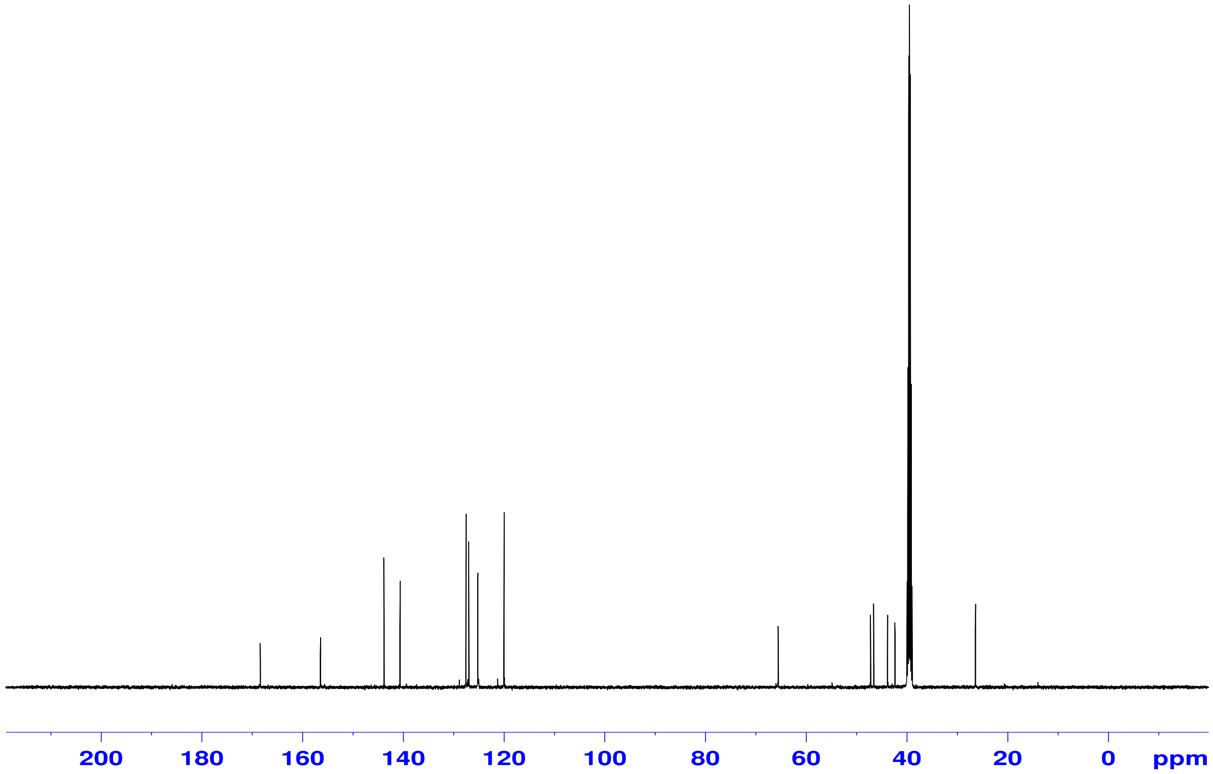

Figure S6. ^{1}H and ^{13}C NMR spectra of **4** in DMSO-d_6.

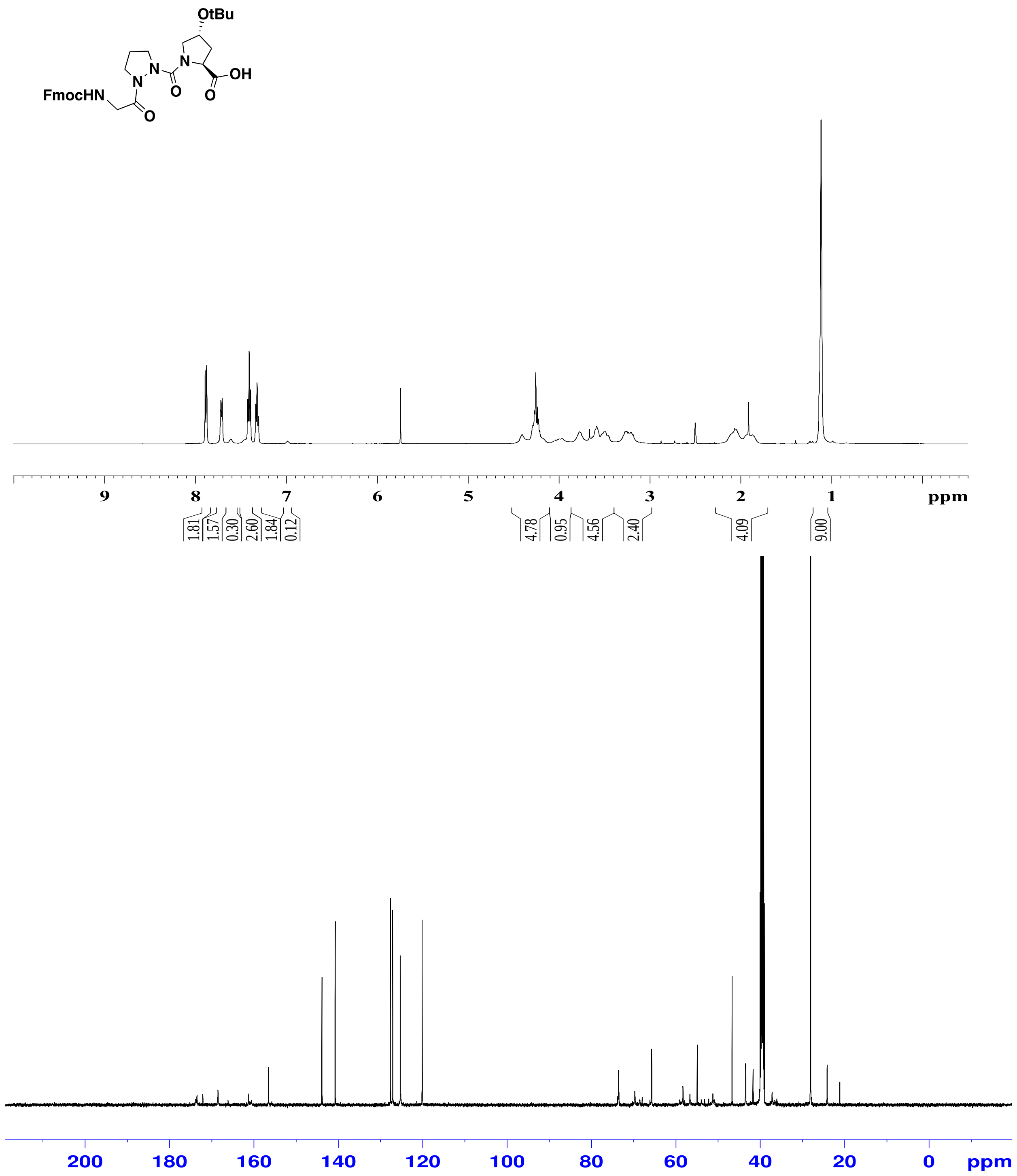

Figure S7. ^{1}H and ^{13}C NMR spectra of **7** in DMSO-d_6.

HPLC Trace and MALDI-TOF Mass Spectrum of CMP (1)

(**1**) H-(Pro-Hyp-Gly)$_2$-(azPro-Hyp-Gly)-(Pro-Arg-azGly)-(Pro-Hyp-Gly)$_4$-NH$_2$

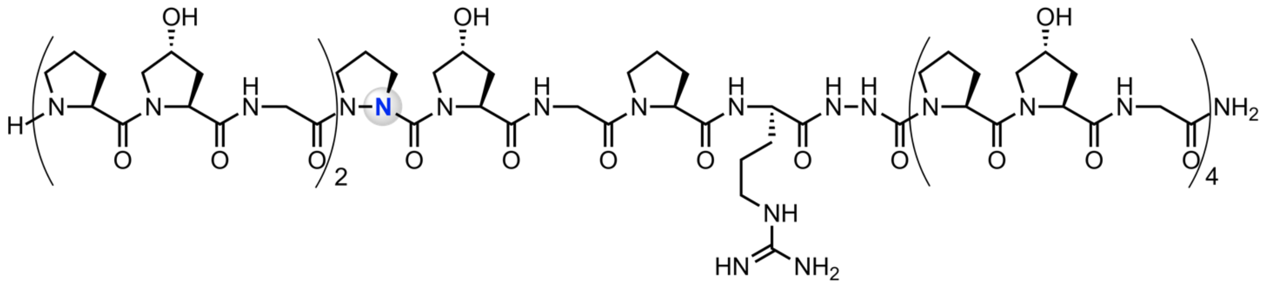

Analytical HPLC: 10-15% ACN/H_2O (H_2O containing 0.1% TFA), 56 min

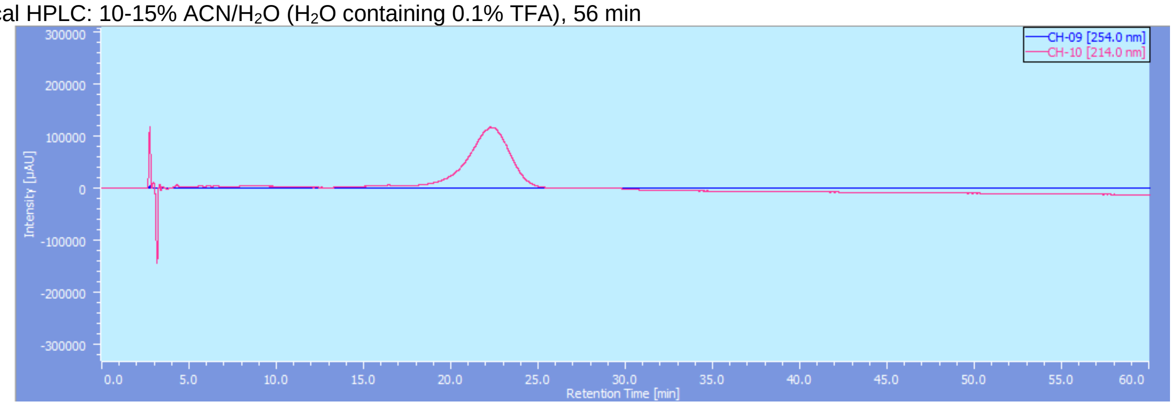

MALDI-TOF MS [M+H$^+$]: Calculated: 2200.053; Found: 2198.721

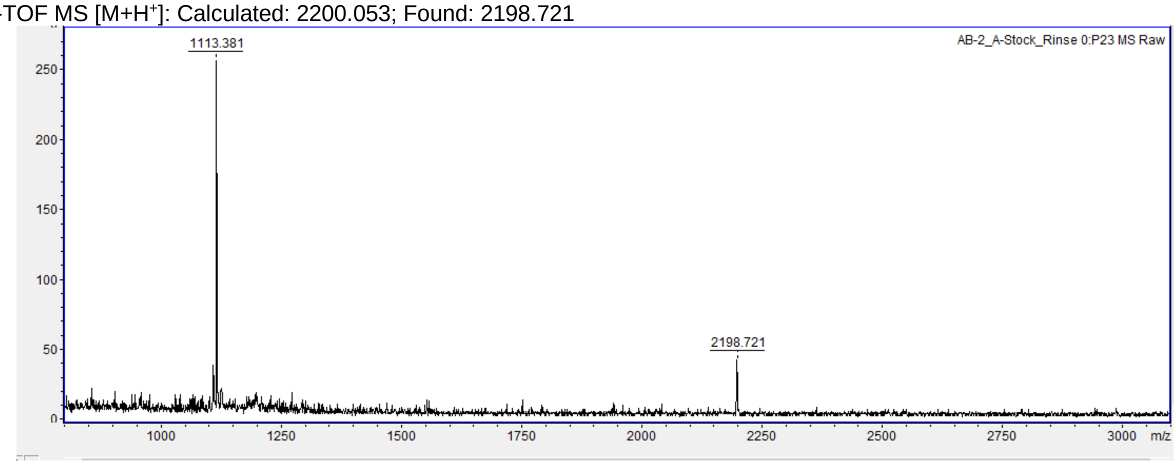

T_m of CMP **1** = 37.2 °C (CD spectroscopy). For reference, T_m of CMP **2** = 50.8 °C.[17]

Crystallization

Peptide **1** was crystallized using sitting-drop vapor diffusion under conditions adapted from Okuyama *et al*.[20] Peptide stock solutions were prepared by dissolving the purified solid product in 18 MΩ H_2O to a final concentration of 8.4 mg/mL (Using UV-vis, measured A_{214} and used extinction coefficient of 60 mM^{-1} cm^{-1}). Crystal trials were prepared by combining 1 μL of the peptide solution with 1 μL of a reservoir solution of 0.1 M Tris-HCl, 30% (w/v) PEG4000, and 0.01 M $Li_2SO_4 \cdot H_2O$ (buffer pH = 7.6). Trays were sealed tightly with plastic tape to create a closed system and prevent solvent evaporation. Trays were incubated at 4 °C. Prior to beamline analysis, crystals were dipped in a drop of a cryoprotectant mixture containing equal volumes of the reservoir solution and 30% (w/v) PEG4000 and then frozen in liquid N_2.

Crystal Structure Refinement

Beamline	APS 24-ID-C
Detector	DECTRIS PILATUS 6M-F
Wavelength (Å)	0.9791
Data collection temperature (K)	100
Resolution range (Å)	48.81 - 1.1 (1.139 - 1.1)
Space group	$P\ 2_1$
Total reflections	39219 (3942)
Unique reflections	19847 (1999)
Unit cell	
(*a*, *b*, *c*; Å)	27.538, 18.175, 48.834
(*α*, *β*, *γ*; °)	90, 91.916, 90
Multiplicity	2.0 (2.0)
Completeness (%)	98.59 (98.76)
Mean I/σ_I	9.92 (1.90)
Wilson B-factor (Å^2)	10.84
R_{merge}	0.03126 (0.4266)
R_{meas}	0.04421 (0.6033)
R_{pim}	0.03126 (0.4266)
$CC_{1/2}$	0.997 (0.627)
CC*	0.999 (0.878)
Reflections used in refinement	19818 (1997)
Reflections used for R_{free}	917 (92)
R_{work}	0.1172 (0.2505)
R_{free}	0.1443 (0.2809)
CC_{work}	0.989 (0.858)
CC_{free}	0.978 (0.920)
Number of non-hydrogen atoms	645
Macromolecules	432
Ligands	45
Solvent	168
Amino acid residues	72
RMSD	
Bonds (Å)	0.032
Angles (°)	2.62
Ramachandran favored (%)	100
Ramachandran allowed (%)	0
Ramachandran outliers (%)	0
Rotamer outliers (%)	0
Clashscore	4.52
Average B-factor (Å^2)	17.78
Macromolecules	14.62
Ligands	15.56
Solvent	26.51

Table S6. Data collection and refinement statistics. Table 1 was generated using Phenix (version dev-3120).[21] Refinement was performed using Phenix (version dev-3126). Manual modeling was performed using *Coot*.[22] Data integration was performed using XDS.[23] Space group validation and data reduction were performed using Pointless (version 1.10.29)[24, 25] and Scala (version 3.3.22),[24] respectively, in the CCP4 suite.[26] Phasing was performed using Phaser.[27]

Calculated Geometries
Calculated geometries with their respective energies (in hartree) and number of imaginary frequencies.

L-3

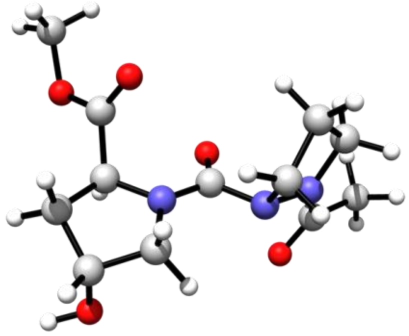

# of imaginary frequencies = 0
E = -1008.779105
0 1
H 0.075384 2.579883 0.838192
H 1.010842 1.681011 2.065708
H 2.715307 3.106681 1.222341
H 3.872489 1.393883 -0.512608
H 3.443312 0.803363 1.112669
H 1.936465 0.346939 -1.508392
H -0.586615 0.316102 2.446295
H -2.241070 0.971280 2.482941
H 4.125405 -3.109662 0.256691
H 4.296392 -3.302968 -1.516019
H 2.754708 -3.733484 -0.712568
H -1.362857 -1.902912 1.879793
H -2.607269 -1.399763 3.048204
H -3.201113 -2.129188 0.397095
H -4.193270 -0.959442 1.305534
H -5.224386 -0.446792 -0.769039
H -4.211288 -1.508540 -1.757885
H -4.847462 0.001486 -2.456209
H 2.787848 3.512168 -1.083943
C 2.231749 2.467424 0.478160
C 3.046533 1.208424 0.175912
C 1.999327 0.247984 -0.420247
C -0.388248 0.048790 -0.127361
C 2.321830 -1.194393 -0.084290
C -1.581531 0.237814 2.013670
C 3.612885 -3.059716 -0.705472
C -2.156687 -1.177388 2.080263
C -3.185673 -1.185376 0.947297
C -3.224411 0.278260 -1.117670
C -4.459946 -0.462796 -1.550314
C 0.916879 1.903158 0.998220
N 0.755841 0.673483 0.207215
N -1.543354 0.516745 0.557434
N -2.719732 -0.085905 0.076207
O -0.432714 -0.832902 -0.992155
O 1.897316 -1.796524 0.882377
O 3.178397 -1.711182 -0.961258
O -2.692721 1.157515 -1.816202
O 1.951180 3.205166 -0.706985

I-3

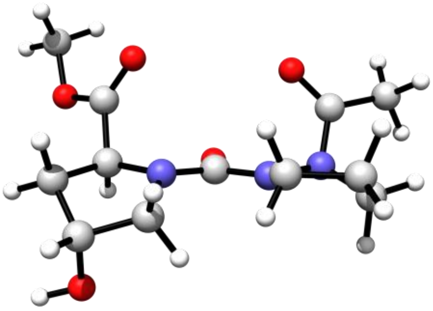

# of imaginary frequencies = 0
E = -1008.769329
0 1
C -2.393727 -2.468930 0.200257
N -0.796721 -0.729295 0.095448
C -3.090118 -1.124301 0.387272
C -2.090794 -0.158474 -0.263007
C 0.299900 -0.160542 -0.503433
C -2.248712 1.254746 0.256413
O 0.169346 0.747544 -1.341921
N 1.530425 -0.614668 -0.123393
O -1.542831 1.789326 1.088416
O -3.306660 1.844627 -0.299734
O 1.919761 -1.446258 1.040738
N 2.660280 0.051487 -0.635218
C -3.601011 3.178392 0.153216
C 3.457222 -1.442615 0.969752
C 3.744933 -0.932281 -0.444917
C 2.861518 1.339903 -0.126649
O 1.998120 1.915310 0.524305
C 4.171349 1.989255 -0.475142
H -0.283169 -2.786257 -0.130138
H -0.698668 -2.300621 1.538017
H -2.775426 -3.240266 0.875257
H -4.073348 -1.087030 -0.085187
H -3.192123 -0.906410 1.455775
H -2.233627 -0.132736 -1.348990
H 1.541856 -1.010913 1.970073
H 1.529397 -2.455413 0.925643
H -3.789036 3.173538 1.228293
H -4.494472 3.479946 -0.389298
H -2.767410 3.842209 -0.081783
H 3.872226 -0.740232 1.698007
H 3.874891 -2.433038 1.154016
H 4.719706 -0.463336 -0.556714
H 3.632159 -1.715351 -1.196729
H 4.565426 1.647722 -1.432901
H 4.899093 1.751369 0.308257
H 4.023634 3.069244 -0.491819
C -0.929866 -2.152227 0.480216
O -2.480239 -2.906586 -1.154399
H -3.413620 -3.026859 -1.379266

D-3

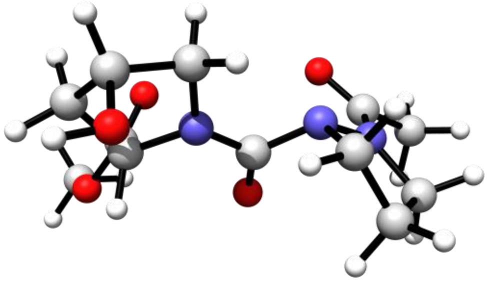

# of imaginary frequencies = 0
E = -1008.777292
0 1
C -2.483875 -2.184416 0.846106
N -0.822477 -0.747973 0.078213
C -3.160675 -0.888165 0.401524
C -2.066729 -0.149552 -0.400382
C 0.347759 -0.183165 -0.266456
C -2.104227 1.336209 -0.087548
O 0.417472 0.810850 -0.995495
N 1.493701 -0.808407 0.314036
O -1.674287 1.827773 0.937971
O -2.724925 2.025968 -1.039435
C 1.861886 -2.107711 -0.306250
N 2.647957 -0.018448 0.148697
C -2.883672 3.436728 -0.801364
C 2.750745 -1.703970 -1.483023
C 3.526019 -0.506660 -0.934245
C 2.765818 1.133666 0.835003
O 1.890330 1.514868 1.631108
C 4.005977 1.935453 0.551066
H -0.359944 -2.582370 1.072418
H -0.982009 -1.279915 2.120007
H -2.959228 -2.619250 1.729769
H -4.056726 -1.066529 -0.195175
H -3.434311 -0.309263 1.288780
H -2.173935 -0.291524 -1.478672
H 2.407335 -2.697145 0.434240
H 0.965481 -2.645833 -0.611699
H -3.477900 3.595971 0.100100
H -3.403614 3.821619 -1.675944
H -1.904760 3.907455 -0.697480
H 3.411185 -2.511563 -1.800553
H 2.128221 -1.398793 -2.328366
H 4.491363 -0.800227 -0.513121
H 3.681139 0.274292 -1.682141
H 3.921250 2.394370 -0.439656
H 4.902228 1.310478 0.560161
H 4.093920 2.720977 1.300876
C -1.057417 -1.747024 1.131990
O -2.428661 -3.137145 -0.211741
H -3.333037 -3.365814 -0.467955

TS1

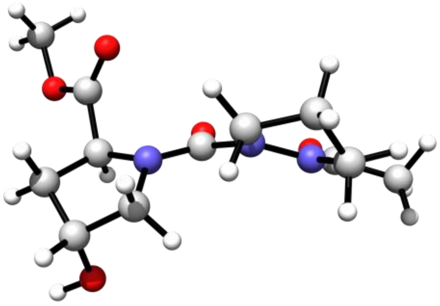

# of imaginary frequencies = 1
E = -1008.745847
0 1
C 1.964652 2.579480 -0.435498
N 0.772952 0.617647 0.087209
C 2.958865 1.457373 -0.147050
C 2.096690 0.207862 -0.373955
C -0.259826 -0.241862 -0.188994
C 2.611418 -0.996647 0.384324
O -0.048904 -1.247648 -0.876197
N -1.452256 0.041003 0.456476
O 2.142958 -1.437282 1.415633
O 3.683574 -1.510310 -0.217467
C -1.506263 0.813743 1.714867
N -2.788954 -0.145912 -0.026080
C 4.293780 -2.641960 0.429515
C -2.927820 0.588594 2.188911
C -3.675711 0.624890 0.869568
C -3.261154 -1.013867 -0.954874
O -2.579124 -1.821254 -1.597358
C -4.750929 -0.923720 -1.213809
H -0.199153 2.483475 -0.244858
H 0.702668 2.375116 1.294452
H 2.286859 3.543938 -0.032546
H 3.832415 1.482220 -0.800808
H 3.285770 1.516346 0.896405
H 2.078529 -0.052689 -1.437258
H -0.743280 0.437657 2.397564
H -1.353709 1.880223 1.539926
H 4.644529 -2.357323 1.423072
H 5.131954 -2.924014 -0.203808
H 3.574979 -3.459805 0.502102
H -3.039462 -0.390899 2.661179
H -3.257953 1.369376 2.874247
H -4.651752 0.153146 0.926371
H -3.779281 1.648546 0.494630
H -5.106257 0.105750 -1.286522
H -5.304168 -1.427320 -0.415041
H -4.943793 -1.441721 -2.152678
C 0.694166 2.084525 0.240272
O 1.697306 2.684658 -1.831218
H 2.525213 2.893026 -2.286454

TS2

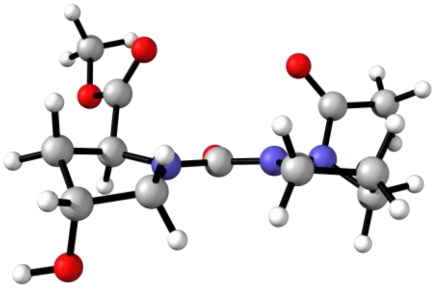

# of imaginary frequencies = 1
E = -1008.768612
0 1
H 0.558594 2.885861 -0.151568
H 0.811368 2.257213 1.503338
H 2.993586 3.067944 1.024093
H 4.179838 0.852904 0.061628
H 3.171039 0.692798 1.525835
H 2.383290 0.044212 -1.359171
H -1.476636 1.677369 1.802583
H -1.441489 2.692409 0.334664
H 3.393526 -3.504362 1.037811
H 3.735812 -3.961351 -0.660154
H 2.051015 -3.970482 -0.052828
H -3.804778 1.224893 1.531707
H -3.778183 2.790698 0.681724
H -4.630428 0.585095 -0.671411
H -3.441789 1.630725 -1.485238
H -4.562999 -1.593264 -1.337012
H -4.986551 -1.484876 0.383646
H -4.180107 -2.941930 -0.228511
H 3.774298 2.910460 -1.183394
C 2.600906 2.360269 0.288709
C 3.169798 0.955138 0.462615
C 2.149110 0.092018 -0.290970
C -0.246397 0.161958 -0.477719
C 2.120854 -1.322748 0.252806
C -1.841471 1.782213 0.779062
C 3.025461 -3.483168 0.010776
C -3.372607 1.778447 0.693070
C -3.633980 1.014568 -0.603666
C -2.900411 -1.276389 0.020439
C -4.242890 -1.858416 -0.328496
C 1.099441 2.157339 0.453602
N 0.888953 0.783459 -0.042161
N -1.460776 0.604399 -0.038086
N -2.607442 -0.040120 -0.534260
O -0.156957 -0.839728 -1.214854
O 1.495978 -1.682044 1.232023
O 2.920165 -2.123530 -0.448387
O -2.087355 -1.883614 0.712768
O 2.820490 2.846683 -1.033746

References

1. Y. Zhang, M. Herling and D. M. Chenoweth, *J. Am. Chem. Soc.*, 2016, **138**, 9751-9754.
2. Y. Zhang, R. M. Malamakal and D. M. Chenoweth, *J. Am. Chem. Soc.*, 2015, **137**, 12422-12425.
3. Y. Zhang, R. M. Malamakal and D. M. Chenoweth, *Angew. Chem. Int. Ed.*, 2015, **54**, 10826-10832.
4. L. A. Carpino and G. Y. Han, *J. Org. Chem.*, 1972, **37**, 3404-3409.
5. M. J. Frisch, G. W. Trucks, H. B. Schlegel, G. E. Scuseria, M. A. Robb, J. R. Cheeseman, G. Scalmani, V. Barone, B. Mennucci, G. A. Petersson, H. Nakatsuji, M. Caricato, X. Li, H. P. Hratchian, A. F. Izmaylov, J. Bloino, G. Zheng, J. L. Sonnenberg, M. Hada, M. Ehara, K. Toyota, R. Fukuda, J. Hasegawa, M. Ishida, T. Nakajima, Y. Honda, O. Kitao, H. Nakai, T. Vreven, J. A. Montgomery, Jr., J. E. Peralta, F. Ogliaro, M. Bearpark, J. J. Heyd, E. Brothers, K. N. Kudin, V. N. Staroverov, R. Kobayashi, J. Normand, K. Raghavachari, A. Rendell, J. C. Burant, S. S. Iyengar, J. Tomasi, M. Cossi, N. Rega, M. J. Millam, M. Klene, J. E. Knox, J. B. Cross, V. Bakken, C. Adamo, J. Jaramillo, R. Gomperts, R. E. Stratmann, O. Yazyev, A. J. Austin, R. Cammi, C. Pomelli, J. W. Ochterski, R. L. Martin, K. Morokuma, V. G. Zakrzewski, G. A. Voth, P. Salvador, J. J. Dannenberg, S. Dapprich, A. D. Daniels, O. Farkas, J. B. Foresman, J. V. Ortiz, J. Cioslowski and D. J. Fox, *Journal*, 2009.
6. Y. Zhao and D. G. Truhlar, *Theor. Chem. Acc.*, 2008, **120**, 215–241.
7. Y. Zhao and D. G. Truhlar, *Acc. Chem. Res.*, 2008, **41**, 157-167.
8. A. V. Marenich, C. J. Cramer and D. G. Truhlar, *J. Phys. Chem. B*, 2009, **113**, 6378–6396.
9. E. F. Pettersen, T. D. Goddard, C. C. Huang, G. S. Couch, D. M. Greenblatt, E. C. Meng and T. E. Ferrin, *J. Comput. Chem.*, 2004, **25**, 1605-1612.
10. C. R. Groom, I. J. Bruno, M. P. Lightfoot and S. C. Ward, *Acta Crystallogr. Sect. B: Struct. Sci.*, 2016, **72**, 171-179.
11. C. F. Macrae, P. R. Edgington, P. McCabe, E. Pidcock, G. P. Shields, R. Taylor, M. Towler and J. v. d. Streek, *J. Appl. Crystallogr.*, 2006, **39**, 453-457.
12. C. F. Macrae, I. J. Bruno, J. A. Chisholm, P. R. Edgington, P. McCabe, E. Pidcock, L. Rodriguez-Monge, R. Taylor, J. v. d. Streek and P. A. Wood, *J. Appl. Crystallogr.*, 2008, **41**, 466-470.
13. Y. Otani, O. Nagae, Y. Naruse, S. Inagaki, M. Ohno, K. Yamaguchi, G. Yamamoto, M. Uchiyama and T. Ohwada, *J. Am. Chem. Soc.*, 2003, **125**, 15191-15199.
14. M. Szostak and J. Aubé, *Chem. Rev.*, 2013, **113**, 5701-5765.
15. Y. K. Kang and B. J. Byun, *J. Phys. Chem. B*, 2007, **111**, 5377-5385.
16. I. J. Bruno, J. C. Cole, P. R. Edgington, M. Kessler, C. F. Macrae, P. McCabe, J. Pearson and R. Taylor, *Acta Crystallogr. Sect. B: Struct. Sci.*, 2002, **58**, 389-397.
17. A. J. Kasznel, Y. Zhang, Y. Hai and D. M. Chenoweth, *J. Am. Chem. Soc.*, 2017, **139**, 9427-9430.
18. E. E. Boros, F. Bouvier, S. Randhawa and M. H. Rabinowitz, *J. Heterocycl. Chem.*, 2001, **38**, 613-616.
19. Technical Note: Determination of the Amino Acid Substitution Level via an Fmoc Assay, Rev.2 3/99, Applied Biosystems, https://www.thermofisher.com/document-connect/document-connect.html?url=https%3A%2F%2Fassets.thermofisher.com%2FTFS-Assets%2FLSG%2FTechnical-Notes%2Fcms_040640.pdf&title=QnJvY2h1cmVzICZhbXA7IFNwZWNpZmljYXRpb25zOiBEZXRlcm1pbmF0aW9uIG9mIHRoZSBBbWlubyBBY2lkIFN1YnN0aXR1dGlvbiBMZXZlbCB2aWEgYW4gRm1vYyBBc3NheTogVGVjaG5pY2FsIE5vdGU6IFJldiAy), (accessed April 2019).
20. K. Okuyama, M. Haga, K. Noguchi and T. Tanaka, *Biopolymers*, 2014, **101**, 1000-1009.
21. P. D. Adams, P. V. Afonine, G. Bunkóczi, V. B. Chen, I. W. Davis, N. Echols, J. J. Headd, L.-W. Hung, G. J. Kapral, R. W. Grosse-Kunstleve, A. J. McCoy, N. W. Moriarty, R. Oeffner, R. J. Read, D. C. Richardson, J. S. Richardson, T. C. Terwilliger and P. H. Zwart, *Acta Crystallogr. Sect. D. Biol. Crystallogr.*, 2010, **66**, 213-221.
22. P. Emsley, B. Lohkamp, W. G. Scott and K. Cowtan, *Acta Crystallogr. Sect. D. Biol. Crystallogr.*, 2010, **66**, 486-501.
23. W. Kabsch, *Acta Crystallogr. Sect. D. Biol. Crystallogr.*, 2009, **66**, 125-132.
24. P. R. Evans, *Acta Crystallogr. Sect. D. Biol. Crystallogr.*, 2006, **62**, 72-82.
25. P. R. Evans, *Acta Crystallogr. Sect. D. Biol. Crystallogr.*, 2011, **67**, 282-292.
26. M. D. Winn, C. C. Ballard, K. D. Cowtan, E. J. Dodson, P. Emsley, P. R. Evans, R. M. Keegan, E. B. Krissinel, A. G. W. Leslie, A. McCoy, S. J. McNicholas, G. N. Murshudov, N. S. Pannu, E. A. Potterton, H. R. Powell, R. J. Read, A. Vagin and K. S. Wilson, *Acta Crystallogr. Sect. D. Biol. Crystallogr.*, 2011, **67**, 235-242.
27. A. J. McCoy, R. W. Grosse-Kunstleve, P. D. Adams, M. D. Winn, L. C. Storonia and R. J. Reada, *J. Appl. Crystallogr.*, 2007, **40**, 658-674.

Author Contributions

A. J. Kasznel performed crystal structure data collection and refinement, peptide characterization, and contributed equally to the preparation of the manuscript, figures, and supplementary information.
T. Harris performed computational calculations and analysis and contributed equally to the preparation of the manuscript, figures, and supplementary information.
N. J. Porter performed crystal structure data collection and refinement and contributed to the writing of the supplementary information.
Y. Zhang performed synthesis, purification, and characterization of peptide **1** and Fmoc-GlyAzProHyp(tBu)-OH synthon and contributed to the writing of the supplementary information.
D. M. Chenoweth designed the study and oversaw the experiments as well as the writing of the manuscript and supplementary information.
